# Diet and Mobility in the Corded Ware of Central Europe

**DOI:** 10.1371/journal.pone.0155083

**Published:** 2016-05-25

**Authors:** Karl-Göran Sjögren, T. Douglas Price, Kristian Kristiansen

**Affiliations:** 1 Department of Historical Studies, Göteborg University, Göteborg, Sweden; 2 Laboratory for Archaeological Chemistry, University of Wisconsin-Madison, Madison, United States of America; University at Buffalo, UNITED STATES

## Abstract

Isotopic investigations of two cemetery populations from the Corded Ware Culture in southern Germany reveal new information on the dating of these graves, human diet during this period, and individual mobility. Corded Ware Culture was present across much of temperate Europe ca. 2800–2200 cal. BC and is represented by distinctive artifacts and burial practices. Corded Ware was strongly influenced by the Yamnaya Culture that arose in the steppes of eastern Europe and western Eurasia after 3000 BC, as indicated by recent aDNA research. However, the development of CW on different chronological and spatial scales has to be evaluated. Examination of the CW burials from southern Germany supports an argument for substantial human mobility in this period. Several burials from gravefields and larger samples from two large cemeteries at Lauda-Königshofen "Wöllerspfad" and at Bergheinfeld “Hühnerberg” contributed the human remains for our study of bone and tooth enamel from the Corded Ware Culture. Our results suggest that Corded Ware groups in this region at least were subsisting on a mix of plant and animal foods and were highly mobile, especially the women. We interpret this as indicating a pattern of female exogamy, involving different groups with differing economic strategies.

## The Corded Ware Culture

**Fig 1:**
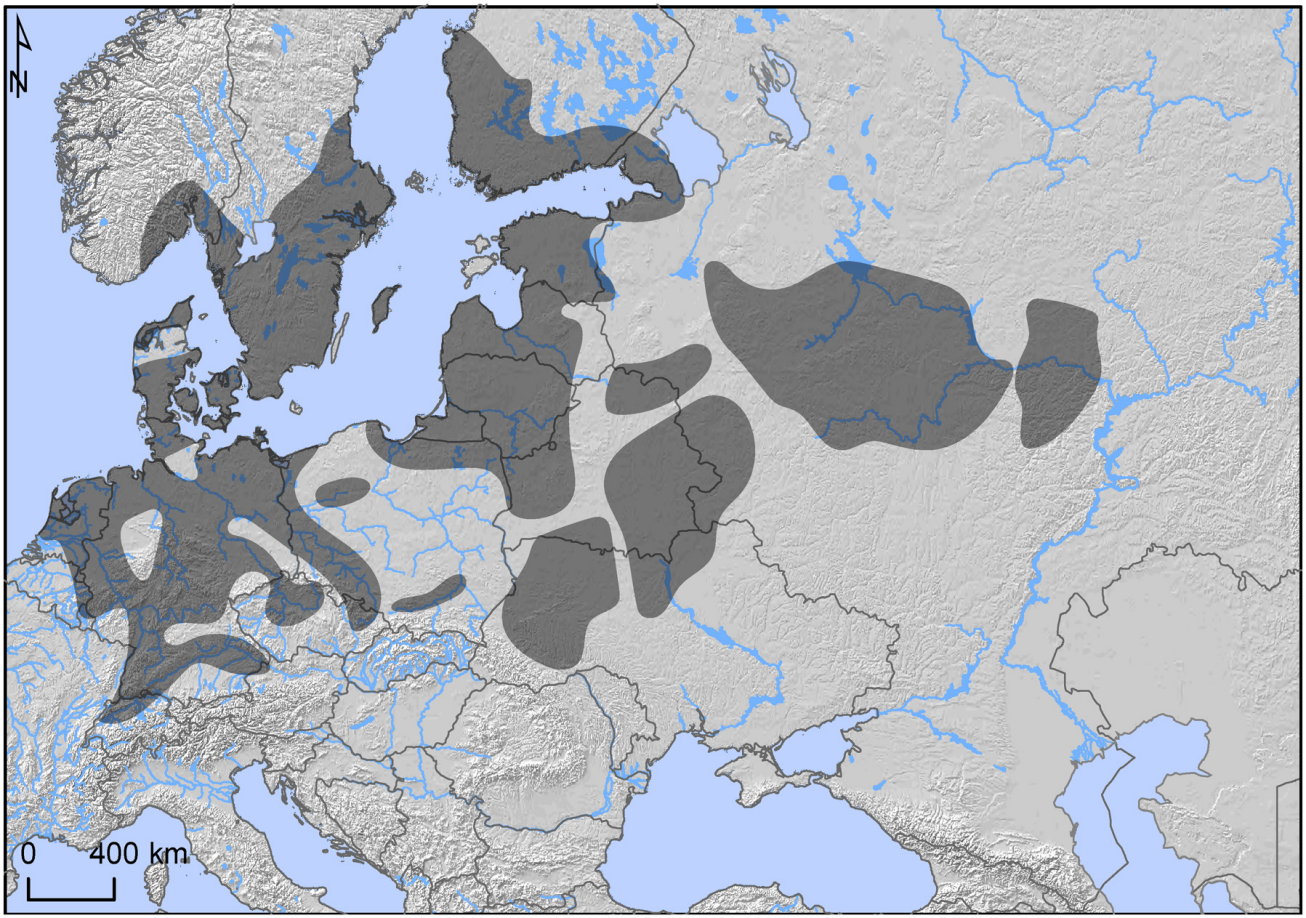
Map of the Corded Ware culture. Redrawn from Müller et al [2], with public domain background data.

The archaeological phenomenon referred to as the Corded Ware (CW) culture is one of the more enigmatic, as well as widely discussed, in European prehistory. Archaeologically it has been defined by a set of material traits, such as cord-ornamented beakers and amphorae, shaft-hole battle axes, and standardised burial practices involving single, sex-differentiated inhumations under barrows, oriented east-west, in contracted (hocker) positions [1]. These burials generally date between ca. 2800–2200 BC and are found over a very large area in central, northern, and eastern Europe (Fig 1). Under the general CW rubric, a number of regionally-defined cultures have been subsumed, such as the Single Grave Culture in Denmark, Holland and N. Germany, the Battle Axe Culture of Sweden, Norway and Finland, and the Fatjanovo Culture in Russia.

The wide geographic distribution and the perceived homogeneity of the culture, coupled with the lack of identified settlements, have given risen to debates regarding the interpretation of this phenomenon. The discussions have concerned among other things the origin of the culture, the mechanism behind its introduction, the identification of a network instead of a mono- or polythetic "culture", the identification of marriage practices, the spread of a common ideology, whether its carriers were also Indo-European speakers, and the nature of settlement and economy.

Regarding the formation of the CW, some archaeologists point out the contribution of different regions to the material set of the "CW-network", while others note similarities with the steppe, in particular with the Yamnaya culture, as a possible area of origin. This is based on similarities in burial rituals. Some authors have suggested that this culture practiced a form of mobile pastoralism, which spread towards the west through migration and/or cultural influence, and gave rise to the CW. In the process, Indo-European language would also have spread over Europe [3, 4, 5, 6, 7]. Recently, these hypotheses have gained support from aDNA studies of Yamnaya and CW burials. Allentoft et al [8] and Haak et al. [9] show that a genetic transformation took place in areas where previous Neolithic DNA was heavily reduced and complemented by Yamnaya DNA. This new genetic presence was lasting and provided much of the genetic material for contemporary European populations. There is increasing evidence for some kind of population reduction or crisis toward the end of the middle Neolithic facilitating this introduction of new genes (e.g. [10]) and recent research has documented the presence of plague among Yamnaya and Corded Ware individuals [11], which may have spread among Neolithic populations prior to the migrations. This needs to be explored in future research.

At the macro-historical level, the old debate over migration versus local adaptation thus seems to be solved. However, we still do not know how migration and other formation processes unfolded in the various regions, and regional variability is evident [12]. Also we have rather scant evidence of the social and economic processes once the Corded Ware Culture was established, but the Eulau cemetery has demonstrated that relations between non Corded Ware and Corded Ware groups could be hostile [13].

Two main propositions have been put forward regarding CW economy. A long-standing tradition has viewed these groups as mobile herders, influenced by the pastoral nomadism ascribed to steppe societies such as the Yamnaya with whom they are likely related [7,8,14]. This view has been mainly supported by negative evidence such as the problems of finding identifiable settlements and house remains in most of the CW regions. This also implies a lack of direct evidence for subsistence (macrofossils, animal bones, etc.). More recently, several authors have argued that CW subsistence economy was a kind of mixed agriculture, not dissimilar to other European Neolithic cultures. These arguments have been based on recent finds of settlements [15, 16,17, 2]. On these sites, both domestic animals and cultivated cereals have been identified. The character and the relative proportion of cultivation vs. husbandry is open to discussion, however. At the Wattendorf settlement in NE Bavaria, for instance, cattle were prominent among the faunal remains, but sheep, goats, pigs and horses were also found. Hunting played a notable role as well, while fish remains were not identified in spite of good preservation [18, 2]. Remains of cereals as well as pulses were also recovered, and agricultural activity at the site is indicated by Late Neolithic colluvium formation on the southern slopes of the hill [2]. Abundant finds of cereals, predominantly emmer, are documented at lakeside settlements from the alpine foreland [19, 20]. At Zürich-Mozartstrasse in Switzerland cattle dominated during the CW phase, and the landscape was more open than in previous periods [21]. A similar opening of the landscape for grazing is also documented from Jutland, Denmark, and more widely in northern Europe [6, 22]. For Schleswig-Holstein and SW Germany on the other hand, detailed pollen sequences suggest a heavily forested landscape [23, 20].

Several of the ideas referred above rely on assumptions regarding human subsistence economy and human mobility, but direct data for these issues have been scarce, with a few exceptions [24, 25, 26]. One of the goals of our project is to examine these questions in the Corded Ware Culture. Were these groups mobile herders or settled farmers, or did they employ a variety of strategies? In this paper, we report a series of new isotope measurements on human skeletal material and discuss their implications for the interpretation of the CW.

Terms like mobility and migration may cover a variety of different processes connected with residential change. Variation may include the spatial extent of movement (short distance/long distance), the temporality (short term/seasonal/permanent), directionality (one-directional/bi-directional/multidirectional), social context of movement (eg. marriage exchange, trade, social gatherings, captivity, flight, or other), or the number of people involved. For the purposes of this paper, we refer to migration as permanent, directional movement of groups of people, while mobility is a general term covering all kinds of movement. More specific interpretations of types of mobility are explained in the text.

## Site Descriptions

**Fig 2:**
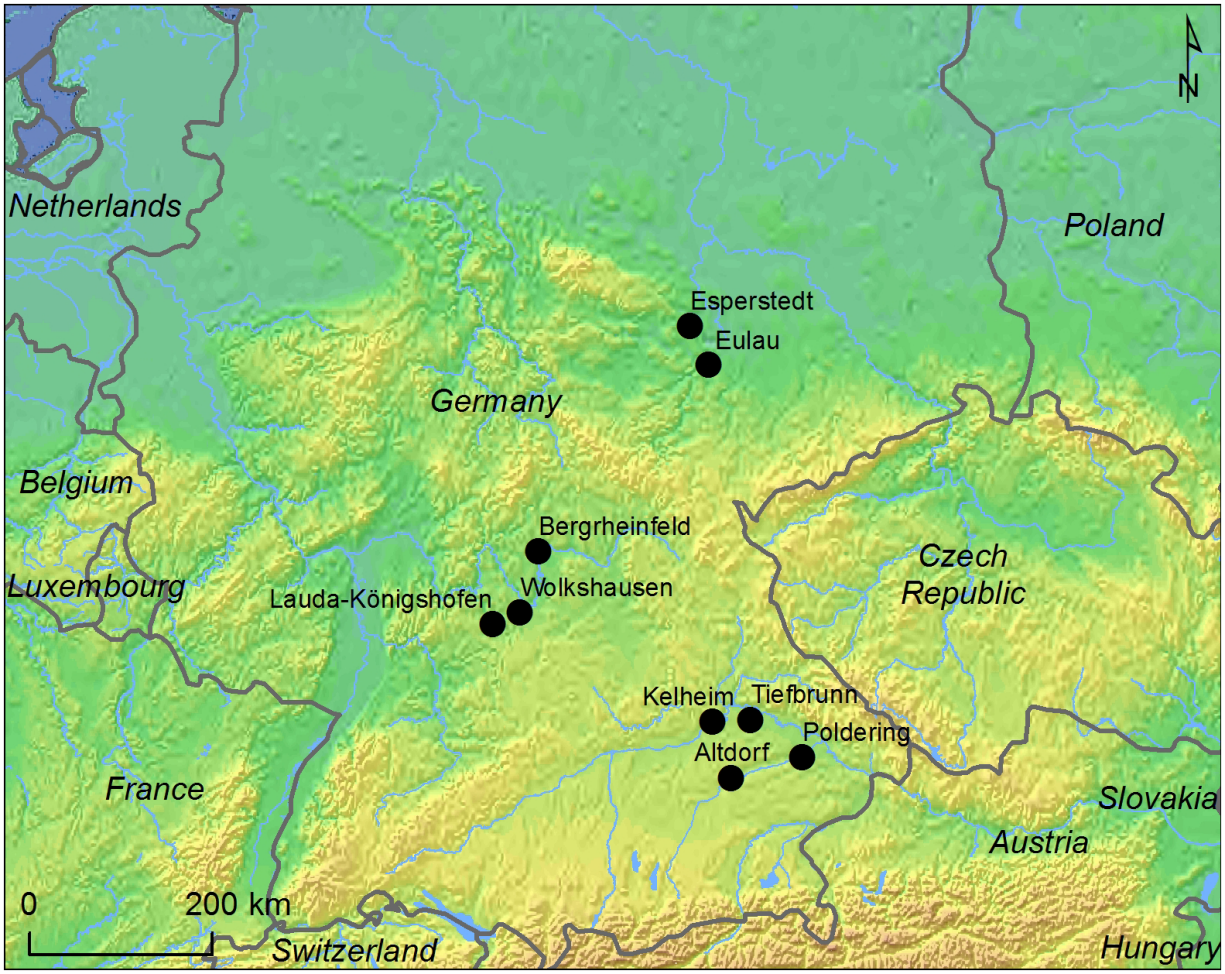
Location of sampled sites and other CW sites mentioned in the text. Map by K-G Sjögren, using public domain data.

Seven sites with CW burials have been sampled for this study. Four are in the Danube basin in SE Bavaria, close to the Danube and the Isar Rivers. Three sites are in NW Bavaria-NE Baden-Württemberg (Fig 2). These sites are located in the Main and Tauber river basins. The sample sites belong to two different regional groups of the CW, namely the South Bavarian group and the Franconian group [27, 28]. The sites are described briefly below.

### Altdorf

The site of Altdorf is in Niederbayern, near the town of Landau an der Isar. It is located some 4 km N of the Isar River (a tributary of the Danube). It was excavated in 2004 in connection with construction activities [29, 30]. One CW grave was found, consisting of an irregular, rectangular grave surrounded by a circular ditch. It contained a single inhumation, buried on the left side with legs contracted, head towards the SE. The skeleton was identified as an adult female, accompanied by a large beaker, a flint blade and a few animal bones. The beaker is classified as a Geiselgasteig type, belonging to a younger phase of the CW. This chronology is supported by new ^14^C dates (S1 Table).

### Bergrheinfeld

Bergrheinfeld “Hühnerberg” is located in the northwest corner of Bavaria near the town of Schweinfurt. The cemetery was excavated 1982–1983 by F. Hoppe and later published by Kerstin Nausch [31]. This cemetery is on loess soil, only 200 m from the shore of the river Main. In the winter 2014/15, a further CW cemetery with 26 graves was excavated just 2 km away, at Bergrheinfeld “Galgenellern”.

The excavations at Bergrheinfeld revealed 31 graves containing 29 skeletons, making this the largest known CW cemetery in Bavaria. Most of the graves were single inhumations in contracted position. Males were oriented to the west, lying on their right side, while females were oriented to the east, lying on their left side, with a single exception. In addition to the single graves, there were two double graves and one grave containing three individuals. Also, six graves lacked a preserved skeleton. Although the cemetery was only partially excavated, most of the graves seem to have been laid out in a circular arrangement around a central grave, number 3. This grave was unfortunately empty but was unusual in that it had a ring ditch with palisade posts.

Anthropological determinations were made by P. Schröter [31]. Nine of the individuals were determined as male, 10 as female, and 10 as children or juveniles. Artifacts were relatively uncommon and consisted of pots, stone axes, bone awls, flint tools, and ornaments made of mussel shells, boar tusks, or fish vertebrae. Pots were only found with females and children, while stone axes were found with adult males. No battle axes were found in any of the graves. Newborn children received no grave goods, while older children and younger juveniles received only pots. During adolescence, a strict differentiation between sexes appeared, seen in body position and the type and placement of grave goods. Differentiation between sexes was in fact visible even in the child graves, however, boys having larger and more highly decorated pots than girls and adult women.

On typological grounds, Bergrheinfeld has been suggested to date from the later phase of the CW (Nausch 1996), but ^14^C dates suggest it belongs to the middle phase (S1 Table). 19 teeth have been analyzed from this site (Table 1). 6 bone samples were analyzed earlier by Asam et al. for collagen and carbonate C, N and O isotopes [24].

**Table 1:** Summary information on sites, sample sizes, and number of isotopic measurements from CW burials in southern Germany. Samples from Asam et al. [24] and Menninger/Trautmann [33, 34] included. N = number of samples, ^14^C = number of dates, etc.

Site	N	^14^C	δ^13^C col	δ^15^N col	δ^13^Cen/c	^87^Sr/^86^Sr	δ^18^Oen/c
Altdorf	1	2	1	1	1	1	1
Bergrheinfeld	25	18	24	24	25	19	25
Kelheim	2	2	2	2	2	1	2
Lauda-Königshofen	25	7	7	7	16	23	16
Poldering	1	1	1	1	1	1	1
Tiefbrunn	5	3	5	5	5	3	5
Wolkshausen	1	1	1	1	1	1	1

### Kelheim

This site is located by the Danube River in Niederbayern, and is located on a former island along the north shore, which has been settled from the early Neolithic onwards. A small group of 3 CW graves was excavated 1978 [32].

A tooth from grave 22 was sampled for our project. This grave contained a single inhumation in left hocker position, oriented ENE, identified as an adult female. She had extensive caries in both upper and lower jaws. In the grave a flint blade, an animal tooth pendant, and a drilled bone plate were found. A radiocarbon date suggests this grave belongs to an early phase of the German CW (S1 Table). Bone samples from graves 22 and 15 were analyzed earlier by Asam et al. for collagen and carbonate C, N and O isotopes [24].

### Lauda-Königshofen

Lauda-Königshofen “Wöllerspfad” is located in the valley of the Tauber in northeastern Baden-Württemberg, not more than 60 km southwest of Bergrheinfeld. The Lauda-Königshofen site was excavated between 1998 and 2000 by C. Oeftiger. An overview and detailed osteological analysis was published by Menninger/Trautmann [33, 34]. The cemetery covered an area of at least 150 x 120 m, but was not completely excavated. In addition to the CW burials, some metal age settlement remains were also found. The geology of the Tauber valley is characterized by loess deposits, and the Lauda-Königshofen cemetery is located on loess soil, on the lower terrace of the river.

A series of CW cemeteries have been excavated in the Tauber valley [35]. There are three large cemeteries known and some 30 smaller sites. The larger ones are Tauberbischofsheim-Dittingheim with 62 individuals, Tauberbischofsheim-Impfingen with 40 individuals, and Lauda-Königshofen with 91 individuals. The cemeteries are dispersed rather regularly along the Tauber valley, on both sides of the river, suggesting a quite densely settled landscape.

**Fig 3:**
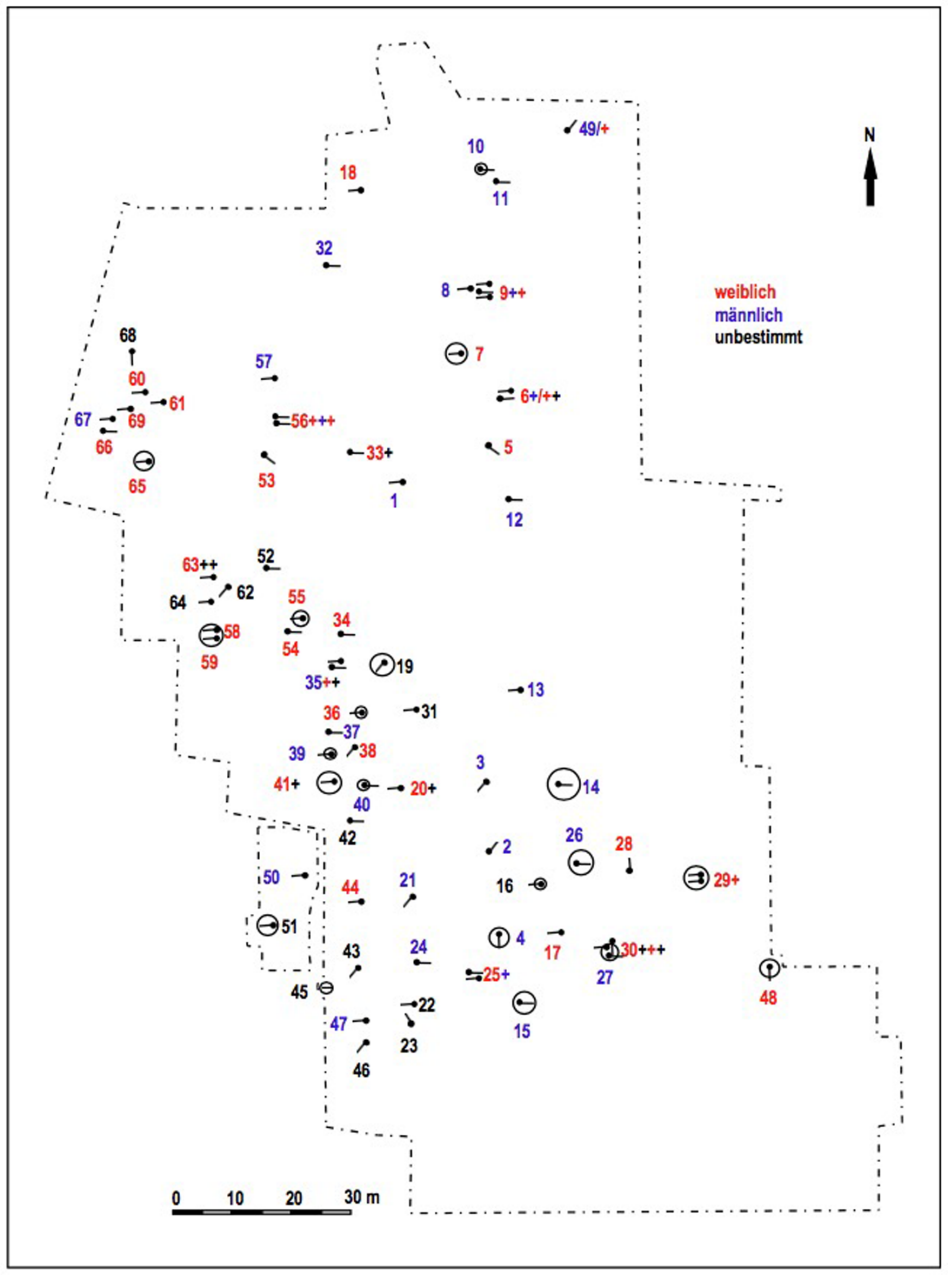
Plan of the Lauda-Königshofen Corded Ware cemetery. Reprinted from [33, 34] under a CC BY license, with permission from Martin Menninger/Trautmann, original copyright 2008.

The Lauda-Königshofen graves consisted mostly of single inhumations in contracted position, usually oriented E-W or NE-SW (Fig 3). A total of 91 individuals were buried in 69 graves. At least 9 double graves and three graves with 3–4 individuals were present. In contrast to the common CW pattern, sexes were not distinguished by body position, only by grave goods. This trait is common in the Tauber valley and suggests a local burial tradition in this area. Stone axes were restricted to males, pottery to females, while other artifacts were common to both sexes. About a third of the graves were surrounded by ring ditches, suggesting palisade enclosures and possibly over-plowed barrows.

18 individuals from Lauda-Königshofen were sampled for our project, seven of which have been dated. The ^14^C dates place the cemetery in the middle part of the German CW, contemporary with Bergrheinfeld (S1 Table). Nine Sr isotope ratio measurements were previously made on teeth from the skeletons at this site [33, 34].

### Poldering

This site is represented by a single grave, excavated in 2004. It is located near the town of Landau an der Isar in the Isar valley, on the upper terrace of the river [29]. The grave was disturbed by plowing, but contained the skeleton of an adult, lying in contracted position on its right side, head oriented to the SW. The grave contained a pot, a flint blade, and a stone axe. ^14^C dating suggests this grave belongs to an early phase of the CW (S1 Table).

### Tiefbrunn

Tiefbrunn is located in the parish of Mintraching near Regensburg, Bavaria. Three graves were investigated in 2001 in connection with road work. Two were undated, but the third (grave 3) contained a triple burial from the CW period [36]. All three individuals were in contracted positions. Osteological analysis suggested that the skeletons belonged to an older male adult and a young adult female, buried on their right sides, and a child ca. 4 years of age, buried on the left side. DNA sexing, on the other hand, indicates both adults were males and the child was a female, making the body positions of the adults consistent with normal CW practice [8]. mtDNA haplogroups were different for all three, indicating that they were not related on the maternal side [8]. Sr isotope ratios suggest that the older man was non-local, while the young man and the child may be locals. The skulls of all three individuals exhibited signs of severe trauma and they had probably suffered violent deaths. The grave thus offers many parallels to the famous Eulau burials in Sachsen-Anhalt [25, 37, 13].

The grave goods consisted of only a flint blade and a hammer-headed bone pin, laid down beside the older male. Such pins are rare in the CW of Central Europe, but common in the Pontic Steppe region where they occur in a variety of forms until they disappear around 2600 BC [38]. All three individuals in the grave were sampled for our project. Bone samples from the two adults were analyzed earlier by Asam et al for collagen and carbonate C, N and O isotopes [24]. The ^14^C dates indicate that the Tiefbrunn grave belongs to an early phase of the German CW (S1 Table).

### Wolkshausen

This site is located near Würzburg in NW Bavaria, only 20 km from Lauda-Königshofen and 40 km from Bergrheinfeld. An Iron Age hillfort was excavated here 1983–1985. During the course of the investigations, nine CW burials were found [31].

One individual from this site was sampled, grave 9. This grave contained an adult male skeleton in contracted position on his right side, head towards the west. The grave goods consisted of a stone axe, a flint flake, a flint blade, a hammer stone, three bone implements, a few pottery sherds, and a few animal bones. 14C datings suggest a rather late date for this grave (S1 Table).

## Samples

Samples of human teeth were collected from burials at the sites described above. Summary data for the sites that were sampled is listed in Table 1. Details on analysed samples are given in S1 Table, which also provides further information on the samples, ^14^C dates, and the isotopic measurements. We aimed primarily at getting representative samples from the two large gravefields, and sampled the smaller sites in order to get longer temporal coverage and comparative samples. It should be noted that large gravefields only appear towards the middle phase of the German CW. From Bergrheinfeld, 5 males, 9 females and 5 infants/juveniles were sampled. From Lauda-Köingshofen, 14 males, 12 females and one juvenile were sampled. First molars were sampled whenever possible; second molars were collected when the M1 was not available, M3 was a last resort. The sampling was constrained by availability and quality of teeth. Our investigations concentrated on the two large cemeteries—Bergrheinfeld and Lauda-Königshofen—where the highest numbers of samples were obtained. As noted, the data includes measurements published by Asam et al. [24] and Menninger/Trautmann [33, 34].

Various information was obtained from the teeth. The collagen in the dentin was used for ^14^C dating, carbon and nitrogen isotopes. DNA was extracted from the tooth roots. The tooth enamel was used to measure strontium, carbon, and oxygen isotope ratios. Summary statistics for these data are provided for all sites and for the two largest samples from Bergrheinfeld and Lauda-Königshofen in Table 2.

**Table 2:** Summary statistics of the isotope results from tooth samples.

	δ^13^Ccoll v PDB	δ^15^Ncoll v AIR	δ^13^Cen v PDB	Δ^13^C spacing	^86^Sr/^87^Sr	δ^18^Oen
All sites						
Mean	-19.9	10.8	-13.4	6.5	0.7101	-4.9
Std	0.6	0.7	1.2	1.2	0.0016	0.5
N	32	32	42	32	51	42
Bergrheinfeld						
Mean	-19.7	10.6	-13.2	6.4	0.7097	-4.8
Std	0.4	0.7	0.6	0.8	0.0009	0.3
N	18	18	19	18	19	19
Wöllerspfad						
Mean	-19.8	11.5	-12.9	7.3	0.7099	-5.2
Std	0.1	0.3	1.3	1.8	0.0014	0.5
N	7	7	16	7	25	16

In the following paragraphs the methods of analysis are discussed along with the presentation of the data. The aDNA results have been summarized elsewhere [8]. We begin with the radiocarbon dating of these samples, followed by a summary of the diet information provided by carbon and nitrogen isotope ratios. A section on isotopic proveniencing focuses on strontium and oxygen isotope ratios as measures of geographic variation.

## Radiocarbon Dating

Direct dates of German CW skeletons have so far been rather few [9, 25, 29, 35, 39, 40]. Here we provide 34 new dates from south German CW graves (S1 Table). Dating of the samples, as well as measurement of δ^13^C and δ^15^N, was conducted at the Oxford laboratory using their standard procedures. The Altdorf and Kelheim skeletons were also previously dated. As can be seen in S1 Table, the agreement between old and new dates is good.

As discussed below, some individuals show rather high δ15N values, and a contribution of freshwater fish to the diet is one of the possible explanations. It could therefore be possible that some of the datings are affected by old carbonates in the water (so-called freshwater reservoir effect). To our knowledge, this problem has not been studied for the rivers in question, although it may be suggested that this is less of a problem for river waters than lakes, especially when much of the tributaries run through non-calcareous areas. Further, the actual contribution of freshwater fish to the diet is hard to estimate as other factors may also have contributed to raising the δ15N values. Until further data are at hand, we prefer to leave this question open as a possible limitation for detailed chronological discussions. For the purposes of this paper, however, we believe that the chronological resolution is sufficient.

The analyzed sites can be chronologically grouped into three broad phases, based on the ^14^C dates. An early phase (ca. 2900/2800-2600 BC) includes some of the earliest dates so far from the German CW, although the shape of the calibration curve does not allow more precise dating. The three smaller sites, Kelheim, Poldering and Tiefbrunn, belong to this phase. The two large gravefields at Bergrheinfeld and Lauda-Königshofen both belong to a middle phase, ca 2600–2500 BC. A late phase, ca. 2500–2300, is represented by the two small sites at Altdorf and Wolkshausen. This fits well with the pottery-based typo-chronologies from the regions [27, 28].

Attempts at Bayesian modeling of the Bergrheinfeld dates suggest that a model of uniform cemetery use agrees well with the dates, and that the period of use may have been quite short (within 8–94 years at 91.9% probability). This would correspond to a few generations only. If we assume that about half of the cemetery was excavated, and that it was used for three generations of ca 25 years each, some 20 people per generation could have been buried at the site. This is close to the estimated population size at Wattendorf, where a settlement of some 25 people is estimated to have existed for 3–6 generations [2].

The dates can be compared to the recently published sites of Eulau [25] and Esperstedt in Sachsen-Anhalt [9]. At Eulau, 8 of the dates fall in the range of 2600–2500 BC, i.e., the middle phase of the CW. Of the four individuals analyzed from Esperstedt, one dates to ca. 2600–2500 BC and the other three to after 2500 BC, i.e., the middle and late phases of the CW. Thus, both sites are quite remote from the actual formation of the CW. This qualification applies to most of the sites discussed here.

Our sampled gravefields thus cover the whole CW period, from the early pioneer phase of Keldering, Polheim and Tiefbrunn to the larger and apparently more consolidated gravefields of Bergrheinfeld and Lauda-Köningshofen to the late phase at Altdorf and Wolkshausen.

## Light Isotopes and Diet

As noted in the introduction, the character of CW subsistence economy remains open to different interpretations. Here we discuss the results from stable isotope analysis of human collagen and tooth enamel for further insight into these questions.

The protein collagen is present in the dentine of the tooth root and remodels very little after formation. Isotope measurements of such samples therefore inform us about diet during the formation of the root, i.e., various stages of childhood and adolescence depending on which tooth is analyzed. The formation of the root of the M1 tooth is completed around the age of 10, for M2 about the age of 15 and for M3 about the age of 18 years [41].

Δ^13^C collagen values in temperate Europe are most commonly used to discriminate between marine and terrestrial protein sources. Marine values are generally higher and may reach values at ca -12‰, while animals feeding on terrestrial C3 plants are commonly at ca -21‰. In addition, there is a small trophic level enrichment, ca 1‰. Δ^13^C values may also be influenced by environmental factors, such as density of vegetation cover. In other regions, δ^13^C may be used to infer the consumption of C4 plants such as maize or millet, since these also have elevated values in relation to C3 plants.

The ‘standard model’ for interpreting δ^15^N in collagen assumes a stepwise enrichment of c. 3 ‰ for every trophic level, so that grasses and vegetables would have levels at c. 3 ‰, herbivores at c. 6 ‰ and carnivores at c. 9 ‰ [42]. Marine and freshwater systems have longer food chains and top predators could attain even higher levels.

As noted by several authors [42, 43, 44], collagen δ^13^C and δ^15^N values only provide partial insight into human diet. There are several reasons for this, but one of the main issues is the routing (metabolic selection) of proteins in the food to build collagen, while other dietary components such as fats and carbohydrates contribute little carbon to collagen under normal circumstances, and no nitrogen. Since animal foods are normally richer in proteins than vegetables and the amino acids are more similar to the ones needed by the human body, collagen signatures are also inherently biased towards animal sources.

A further problem is that the trophic level enrichment in humans is insufficiently understood. Initially, a value of 3‰ was proposed, but more recently values up to 5 ‰ have been suggested [42].

That δ^15^N values in cereals can also be raised significantly as a result of cultivation practices such as manuring has been shown experimentally [45, 46]. Obviously, there are serious problems trying to discuss the trophic level of humans in such cases. In addition, there is also a possible environmental influence, at least in arid zones.

**Fig 4:**
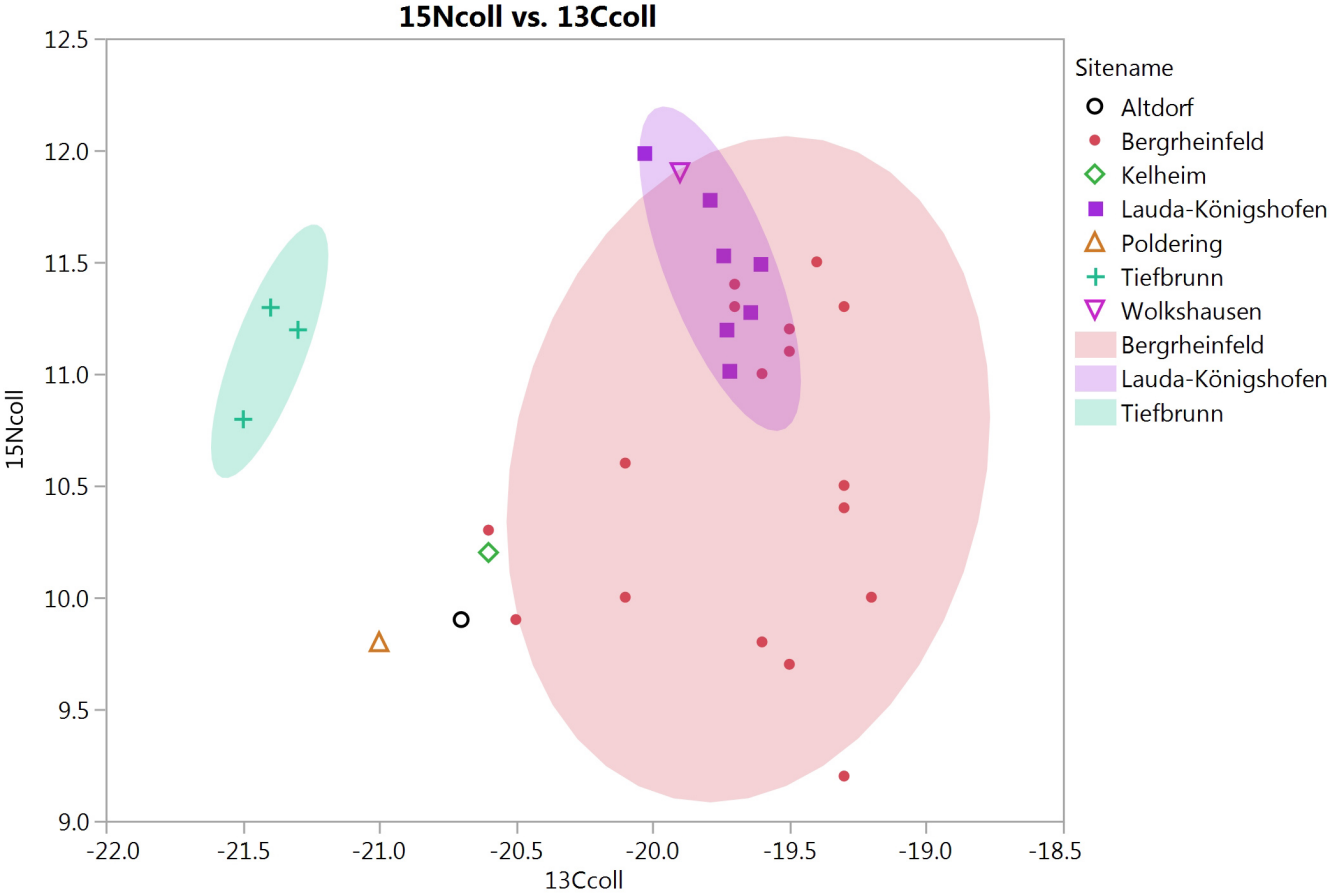
Scatterplot of δ ^15^N vs. δ ^13^C for all samples.

δ^13^C and δ^15^N collagen values are presented in S1 Table and Fig 4. Summary statistics are given in Table 2. Overall, δ^13^C values are consistent with a terrestrial diet based on C3 plants and/or animals feeding on them. No indications of the consumption of C4 plants, e.g., millet, are present. δ^15^N values range from moderate (~9 ‰) to rather high (~12 ‰). According to the “standard model” [42], this could be interpreted as indicating protein sources from a high trophic level, i.e., mainly animal (including milk products). For the highest values, an input of protein from freshwater fish may also be indicated, which is consistent with the location of most of the sites close to rivers. The possible effect of manuring should also be considered, although this aspect remains to be explored in future work.

A second point to make is the high degree of variability in the data, both between and within sites (Fig 4). The individuals from Tiefbrunn have considerably higher δ^13^C values than at other sites, while the two large sites of Bergrheinfeld and Lauda-Königshofen differ particularly in terms of δ^15^N. Individuals from Bergrheinfeld show very high variation in δ^15^N, amounting to almost 3‰, i.e. one whole trophic level according to the standard model.

It can be noted that most of the analyses were made on permanent molars, preferably M1, so that the lactation effect should be minimal. Also, the children analyzed do not show higher δ^15^N than the adults. The variation may instead be due to varying proportions of freshwater fish, animal meat, and vegetables.

It can also be noted that the two large sites are situated rather close to each other and in very similar environments, river valleys covered with loess soils. These data therefore argue against any simple model of CW subsistence. Rather, we see a picture of locally varying mixtures of available food sources.

In terms of comparative data, there is an extensive body of collagen isotope values for various Neolithic periods in southern Germany, although with a clear emphasis on the early Neolithic. Isotope data for Bavaria, including some of the sites discussed here, are reported by Asam et al. [24]. An overview and summary of available data is given in Mörseburg et al. [47].

According to these studies, there is a rise in collagen δ^15^N as well as in carbonate δ^13^C from the early Neolithic to the CW, pointing to a higher trophic level of protein sources in the CW than in earlier periods.

**Fig 5:**
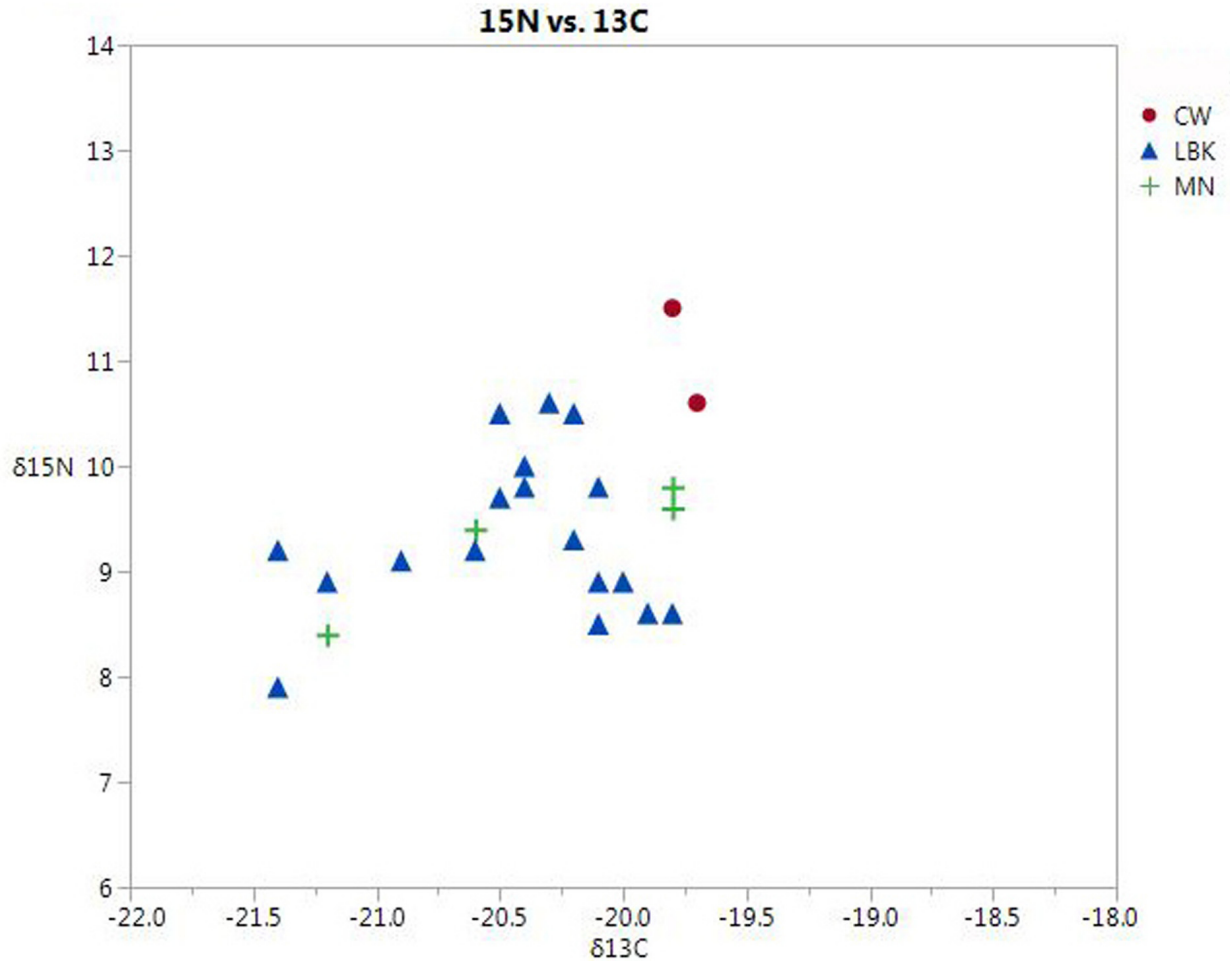
Mean δ13C and δ15N values at Bergrheinfeld and Lauda-Königshofen, compared to early (LBK) and middle Neolithic (Rössen, Hinkelstein, Grossgartach) sites in southern Germany (ca 5500–4100 BC). Comparative data from Mörseburg et al. [47].

This is also supported by our data. In Fig 5, mean values of the samples from Bergrheinfeld and Lauda-Königshofen are plotted against early and middle Neolithic data from southern Germany (mainly the Rhine and Danube valleys). Both CW sites are higher in δ^15^N and δ^13^C than earlier sites. This indicates a shift in protein sources, although part of the explanation may also be environmental, at least for δ^13^C. It should be noted that there is a considerable time gap between the middle Neolithic sites and the CW sites, and it is therefore not possible to say whether this was a rapid shift or a longer term trend.

The shift could originate from a higher proportion of freshwater fish or of animal vs. vegetable protein, from a higher reliance on milk products, from more intense practice of manuring cultivated fields, or from a combination of such factors. It can be noted that studies of weed composition at lakeside CW settlements indicate the practice of intense horticulture, as for other Neolithic periods [48, 19]. Nitrogen isotope ratios in CW cereals are not well known, but one sample from a Single Grave Culture context at Damsbo in Denmark did show elevated δ^15^N values, suggestive of manuring [46, 49]. At present, however, it is not possible to distinguish between these possibilities.

For the CW of Poland and the Baltic countries, collagen isotope data are reported by Eriksson [50], Antanaitis-Jacobs et al [51] and Pospieszny [52]. In these regions, δ^15^N values are even more variable than in Germany, in some cases indicating a significant freshwater fish contribution while in others, such as Lithuania and Estonia, δ^15^N values are lower than in Germany.

Most paleodiet work involving carbon isotopes has focused on organic collagen in bone. As noted above, the view of human diet from collagen is only partial. Carbon is also present in the mineral portion of bone and tooth enamel as carbonate and records the aggregate (protein, fats and carbohydrate) value of individual diet [43, 53, 54]. Although there are potential problems with contamination in apatite [55, 56], it can nonetheless provide substantial insight into the composition of individual diet. Variation in enamel carbonate values can reflect geographic, individual, and/or seasonal fluctuations in the δ^13^C of dietary input.

Since enamel δ^13^C is influenced by the whole diet, the difference between collagen and enamel values should be greater in cases where much of non-protein food components are different from protein sources than in cases where they coincide. This is supported by measurements on animals; carnivores tend to have low collagen-enamel spacing (< ca 4–5 ‰) while herbivores show higher differences [53]. The method has so far not been widely applied to European Neolithic material, but some comparative data are available from south German Neolithic samples [24] and from the Swedish TRB passage grave at Frälsegården [44].

**Fig 6:**
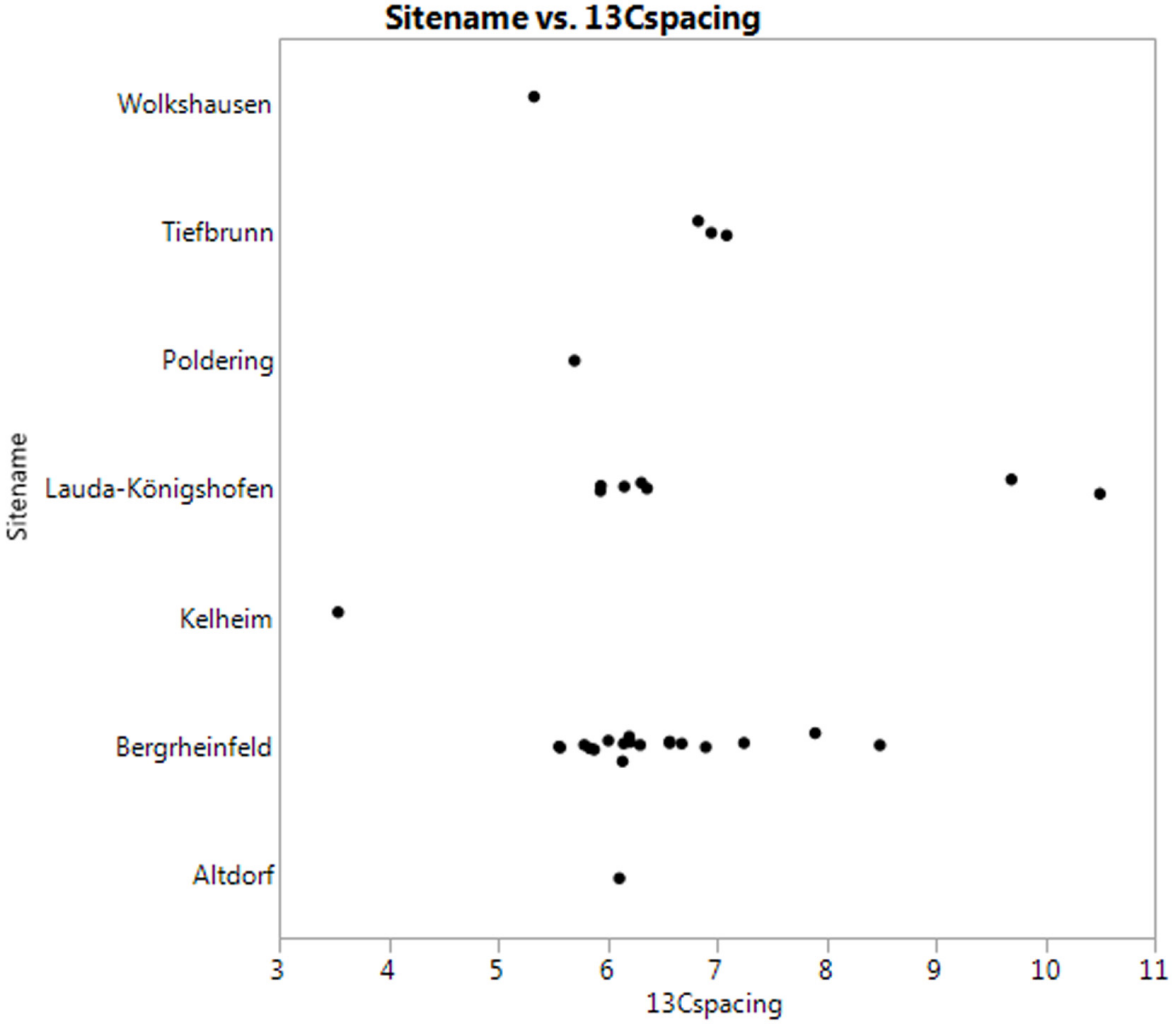
δ13C spacing by site.

In Fig 6, the δ^13^C collagen-enamel spacing (conventionally written as Δ^13^C _col-en_) is shown for the CW samples. Most of the individuals cluster at a spacing of 6–7 ‰. This is comparable to the results for Frälsegården in Sweden, and may indicate a diet with a substantial, but not dominant, vegetable component. There is also a large amount of variation in the values, as was the case for collagen. One individual, a female from Kelheim, has a very low spacing, suggesting high animal food intake during early years. Interestingly, this individual has very high spacing and rather low δ15N in a bone sample (Table 3), indicating high plant food contribution during her final years. This could also explain her high caries frequency.

**Table 3:** Carbon and Nitrogen isotope vales from bone samples at the studied sites. Data from Asam et al. [24].

Site	Context	Sex	Element	δ^15^Ncoll	δ^13^Ccoll	δ^13^Ccarb	δ^13^C coll-carb spacing
Kelheim	grave 15	M	Mt	10.6	-21.39	-14.7	6.69
Kelheim	grave 22	F	fibula	9.82	-21.67	-9.89	11.78
Tiefbrunn	grave 3/1	M	Rib	10.21	-21.67	-12.68	8.99
Tiefbrunn	grave 3/3	M	Rib	9.58	-21.99		
Bergrheinfeld	grave 1 1982	F	Rib	9.43	-20.41		
Bergrheinfeld	grave 1 1983	F	phalanx	9.65	-20.64		
Bergrheinfeld	grave 2 1983	F	fibula	8.97	-20.81		
Bergrheinfeld	grave 5 1983	M	Rib	9.84	-20.69		
Bergrheinfeld	grave 9 1983	M	Rib	11.35	-20.39	-13.03	7.36
Bergrheinfeld	grave 11 1983	M	Rib	11.04	-20.64		

On the other hand, there are also four individuals, two from Bergrheinfeld and two from Lauda-Königshofen, which show very high spacing (7.5–10.5 ‰). Clearly, these individuals have had a different diet from the majority, probably a lower proportion of animal food (including fish).

**Fig 7:**
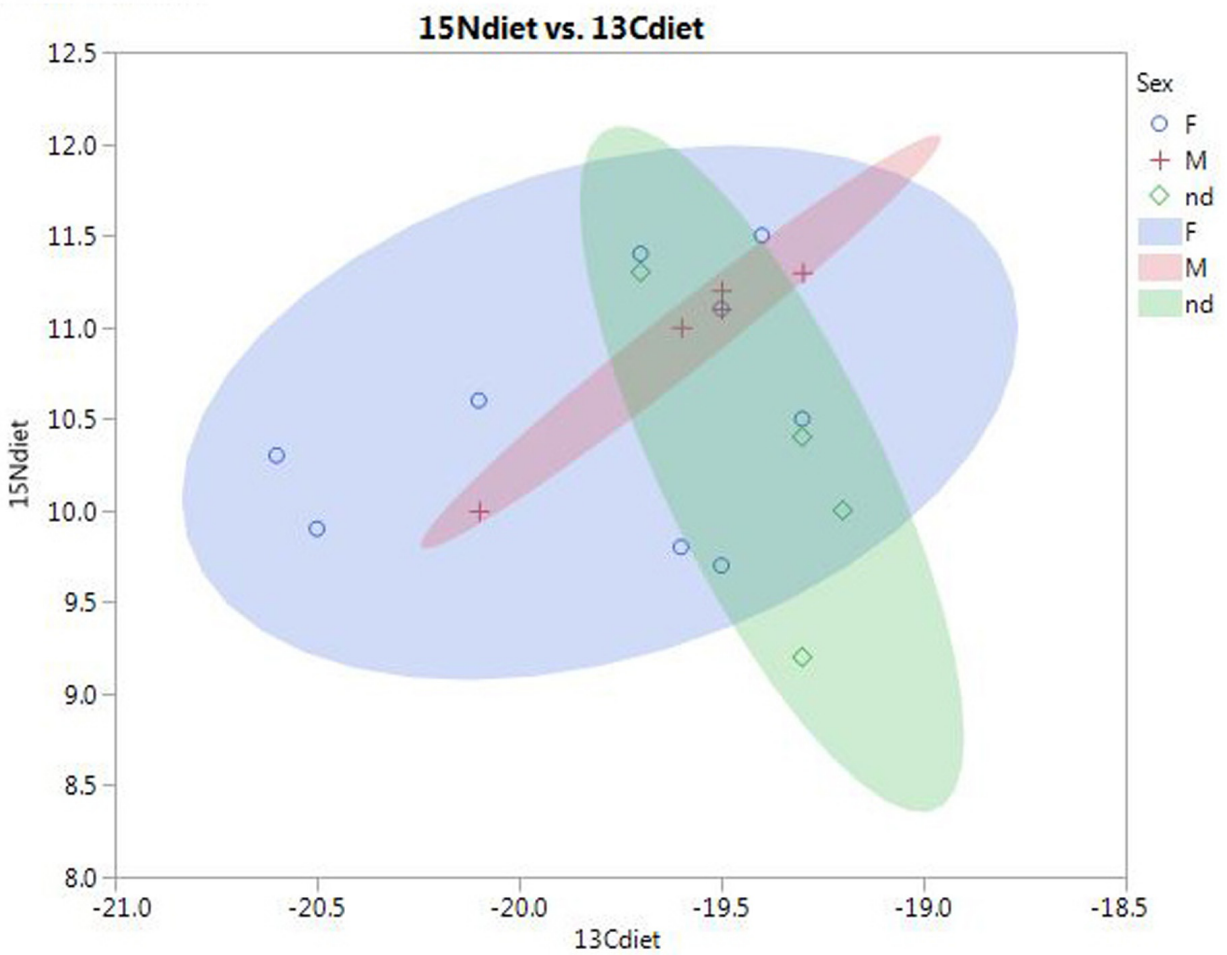
δ^13^C and δ^15^N collagen values for Bergrheinfeld.

Interestingly, dietary isotope values correlate strongly with sex distinctions. Fig 7 plots δ^13^C and δ^15^N collagen values by sex for Bergrheinfeld. The main difference between the sexes is the more restricted and more linear variation for the males, while the females show higher variation.

The individuals of unknown sex (children and juveniles) show another kind of variation than the males.

**Fig 8:**
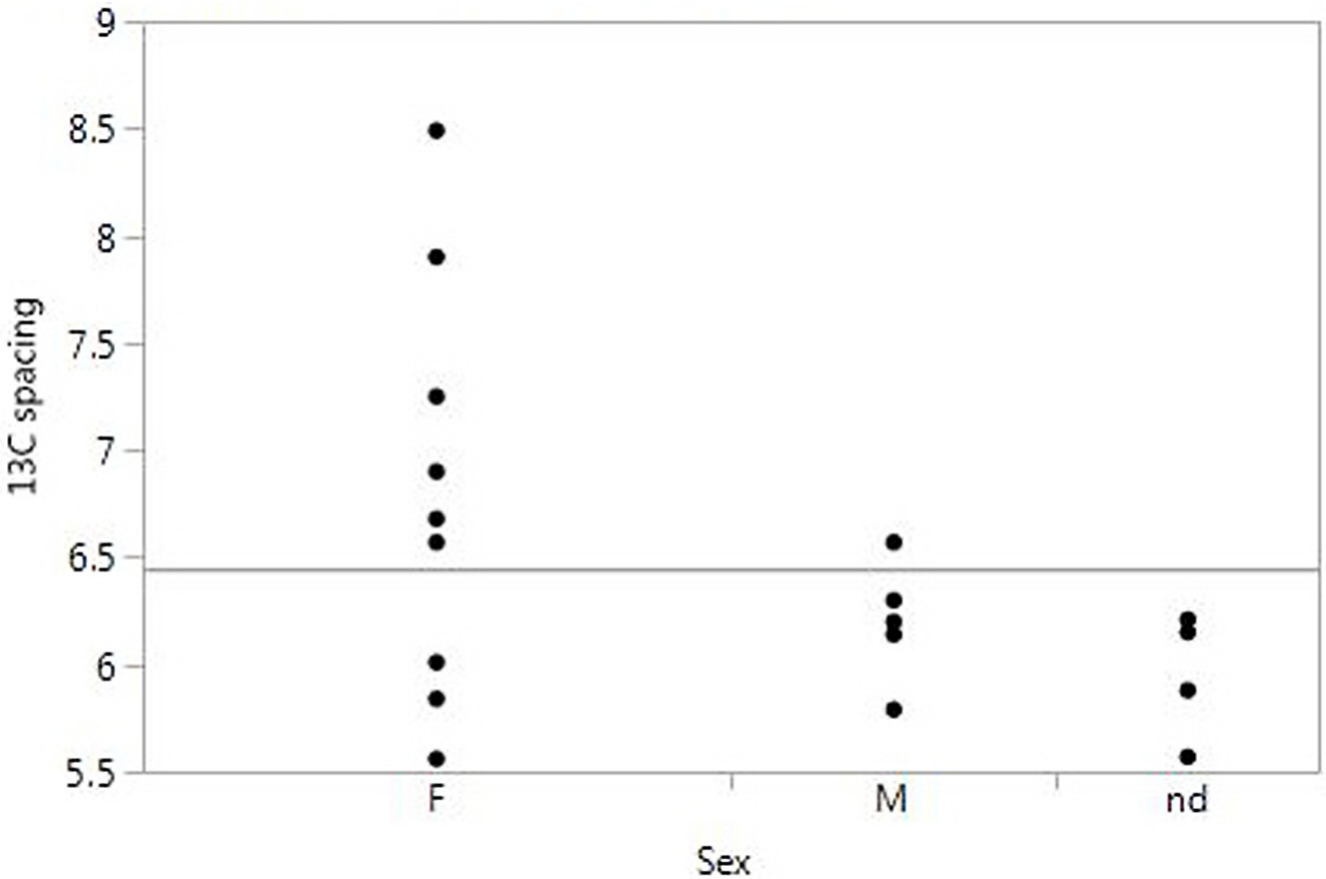
Δ^13^C collagen-enamel spacing by sex for Bergrheinfeld.

The difference between sexes at Bergrheinfeld is further supported by Fig 8, showing δ^13^C collagen-enamel spacing for males and females. The males show a much more restricted range of variation, while the female values also include high values suggesting more vegetable input.

These analyses strongly suggest that there are clear differences in diet between males and females at this site, the males consuming more animal foods and some of the females more vegetable foods. The strict differences in burial treatment is thus paralleled with dietary variation in early life. At Lauda-Königshofen, unfortunately, there are too few values to give a clear picture.

Since these values are from tooth samples, they reflect conditions during early life. The question is whether the dietary distinctions remained constant through life or applied only in childhood and juvenility. The few available bone values (Table 3) suggest that dietary distinctions can be traced also in adult life. For δ^15^N, four out of six male values are above 10‰, while all four female values are below this value. Of the four δ^13^C collagen-carbonate spacing values, the only female has a value at almost 12 ‰ while the three males are at ca 7–9 ‰. Although too few to be conclusive, these data point in the same direction as the values from the teeth.

There would seem to be at least two possible scenarios. One explanation is that females generally had a different diet from males, often involving a larger vegetable consumption. This possibility gains some support from the isotope data reported for adults by Asam et al, cf above. On the other hand, local women at Bergrheinfeld do not differ from local males isotopically, and at least one woman (Kelheim) changed her diet during life. A general difference in diet does not seem probable, which does not exclude however that such practices were at hand in some groups and not in others.

Another possibility is that of variation between settlements or regions, some areas having greater focus on husbandry while other areas put more emphasis on vegetables and cereals. Since many of the incoming women to Bergrheinfeld are also high in δ^13^C spacing, while locally born females and juveniles have a diet similar to that of males, what we may be seeing is a pattern of exchange (for instance marriage exchange) between sites or regions with differing emphasis in their subsistence production.

One question that arises here is whether such exchange would have involved only CW groups or if people moved between different cultural spheres. The latter seems to have been the case at Eulau where incoming women probably originated in the Schönfeld group somewhat further north. Contacts between these groups is also supported by Schönfeld pottery found in CW graves in the Elbe-Saale area [39].

For our region we are not in a position to answer this question with any confidence, unfortunately. Within our two CW regional groups, where the longest distance between them is around 200 kilometers, we witness the same diet among locals. However, the analyzed sites are all in river valleys covered by loess soils, having low Sr isotope ratios. Higher values can be found in more upland situations in southern Germany, some distance away from the river systems. These areas are at present not very well-known archaeologically. We can therefore not say whether they were occupied by CW groups or of other cultural groups.

One possibility could be that incomers originated in late survivors of earlier Neolithic groups, such as Bernburg, Wartberg, Cham or Goldberg III. At present knowledge, however, these are probably not dated later than 2700 BC [57, 58, 59]. A further possibility is that of an origin in the core CW region of Elbe-Saale, some 100 km to the north of Bergrheinfeld. Recent baseline investigations have shown this area to be rather varied isotopically, and to include values between 0.710 and 0.712, matching some of the non-locals at Bergrheinfeld and Lauda-Königshofen [60]. Bell Beaker groups on the other hand do not appear in this region before 2500 BC [61].

At Bergrheinfeld, there is nothing in the burial practice or artifact associations that distinguishes incoming women from other people buried there, or from the CW burial customs in general. The one exception is a non-local female from the double grave 8, who was buried on her right side facing north. This is certainly an exceptional position, but would also be exceptional in other Neolithic contexts, and may probably have another explanation than cultural background. Three graves, (9, 11 and 20) are oriented more N-S than E-W and are suggested to show Bell Beaker influence [31]. Only grave 9, a female adult, has been analysed here. The result indicates that she was a local. For the Lauda-Königshofen cemetery, it is hard to evaluate this question since no detailed archaeological description has been published.

If non-local women originated in non-CW groups, they would therefore have had to adopt local customs and be accepted as belonging to the local society, their identity as CW being affirmed at the time of burial. In sum, we need more comparative evidence from a wider area before we can safely evaluate the origin of the incoming women.

As may be seen in S1 Table, there is no clear trend over time for these samples. Thus there are no indications of a significant change in diet during the course of the CW. As data for the early and late phases of the CW are still few, this needs to be checked with further analyses.

Comparing the Sr data with δ^13^C spacing gives interesting, but different results for the two sites. At Bergrheinfeld the local individuals are also quite homogenous regarding diet; the δ^13^C spacing clustering around 6 ‰. The non-local individuals on the other hand have a more varied diet, and include also all the individuals with higher spacing, i.e., more vegetables in their diet. As noted above, these individuals are all women.

At Lauda-Königshofen, this seems not to be the case. Here, the two outliers with a very high δ^13^C spacing both have Sr values around 0.709, consistent with local residence during childhood. This does not exclude however, that they could in reality have spent their childhoods in another area with a Sr signature similar to that in Lauda-Königshofen.

Finally, we note that the diet results from isotope analysis are in some contradiction to the conclusions drawn by Menninger/Trautmann [33, 34] for Lauda-Königshofen. Based on osteology and trace element data, he suggested a diet based on high meat/milk intake, as well as a mobile herding lifestyle. While suggestive, the osteological data presented by Menninger may often be given several interpretations, and trace element analysis is riddled with problems of contamination and unknown variation [58]. The methods used here offer more direct insights into these questions, and should be given greater weight.

## Isotopic Proveniencing

In the present study we focus on human burials from the two large Corded Ware cemeteries in southern Germany. We use strontium and oxygen isotopes in human tooth enamel to investigate past mobility. In the following pages we outline the basic principles of isotopic proveniencing and discuss the background values that might be expected to occur in the region. Prior investigations have provided useful information on expected isotopic ratios in the study area. We discuss background strontium isotope ratios in southwestern Germany (Baden-Württemberg) and Bavaria separately.

### Strontium Isotopes in Enamel Apatite

Strontium isotope analysis is a robust means for examining past mobility. The principle is straightforward. Strontium moves into humans from rocks and sediment through the food chain and is deposited in the human skeleton [62, 63, 64, 65]. While our bones experience continuous chemical and structural turnover during life, the enamel in our teeth forms in infancy and early childhood and remains unchanged through life. Thus enamel records the isotopic signal of early childhood. If an individual moves to a new location in a different geologic context, or is buried in a new place, the enamel isotopes will differ from those of the new location, registering that individual as non-local.

The strontium isotope ratio (^87^Sr/^86^Sr) in rock and sediment depends on their age and composition. The heavier isotope (^87^Sr) is formed from the radioactive decay of rubidium-87. Thus, older rocks with more rubidium have a higher ^87^Sr/^86^Sr ratio, while younger rocks with less rubidium are at the opposite end of the range with lower ratios [66]. The proportion of ^87^Sr varies in the terrestrial ecosystem, but averages around 7% of total strontium, and ^86^Sr is about 10%. Their ratio normally varies from about 0.700 in rocks with low Rb to 0.730 and much higher in high-Rb rocks that are billions of years old. Values in human tooth enamel range from approximately 0.705 to 0.725. Sediments reflect the ratio of their parent material.

The application of isotopic proveniencing to ancient human teeth has become standard practice in archaeology. There are several published summaries of the method [66, 64, 67]. Analytical methods are described in detail in a number of publications [68, 69, 70, 67], see also S1 File. Numerous examples of the application of strontium isotope ratios to a variety of archaeological questions have been published [71, 72, 73, 74, 75].

An essential question regarding strontium isotope analysis concerns the local strontium isotope signal for the area in which human or faunal remains are found. In actual fact, levels of strontium isotopes may vary from the geological background for a number of reasons [76, 77, 78]. Factors include differential weathering of minerals in rock, the deposition of aeolian, alluvial, or glacial sediments on top of bedrock geology. New ratios may also be introduced by salt spray or rainfall in coastal areas or atmospheric dust in some regions. For this reason, it is necessary to measure *bioavailable* levels of ^87^Sr/^86^Sr to ascertain local strontium isotope ratios [77, 78].

Bioavailable strontium isotope ratios are those values actually present in the local food chain. The bioavailable isotopic signal of the place of burial can be determined in several ways, including the bones of other archaeological fauna at the site or from modern fauna in the vicinity. This baseline information on isotope values across an area needs to be obtained in order to make useful and reliable statements about the origins of the materials under study [77, 69] Bioavailable baseline information from southern Germany (Baden-Württemberg and Bavaria) is discussed in the following paragraphs.

#### Baden-Württemberg

**Fig 9:**
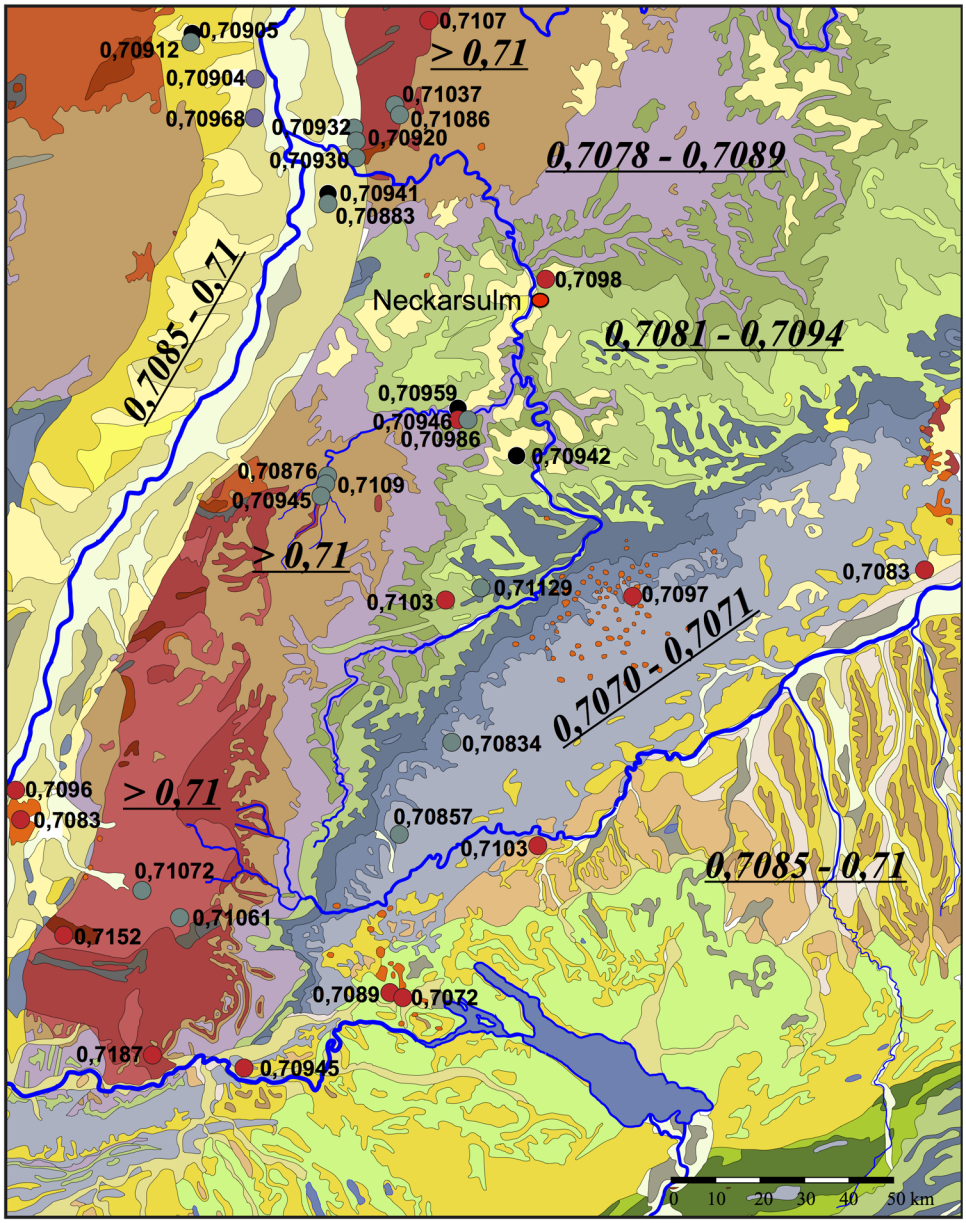
The geology and 87Sr/86Sr values for Baden-Württemberg, southwest Germany. Modified after [118].

A number of studies of strontium isotopes and past mobility have been conducted in southern Germany in the last 20 years, providing a good background for baseline bioavailable ^87^Sr/^86^Sr. South-western Germany, here regarded as the state of Baden-Württemberg, is a geologically diverse region and includes a variety of sedimentary, igneous, and metamorphic formations, largely of Mesozoic age (Fig 9). In addition, there are alluvial, glacial, and loess deposits on top of bedrock in the region.

There are a number of major geological units in Southwestern Germany. Older gneiss and granites dominate the higher elevations of the Black Forest and Odenwald to the east of the Rhine. The Buntsandstein is a red sandstone from the Lower Triassic that outcrops at the base of these highlands. Another upland zone, the Swabian Alb, composed of Jurassic limestones, runs northeast-southwest across the area largely to the north of the Danube. To the south of the river, deep deposits of Tertiary molasse—a sandstone derived detritus from the formation of the Alps—are found along with younger glacial and alluvial sediments.

The central part of the study area, east of the Black Forest and north of the Swabian Alb, is dominated by the Muschelkalk and Triassic Keuper deposits, overlain by fluvial sediments and loess. The Muschelkalk is a Middle Triassic shell limestone and dolomite that lies against the eastern slopes of Black Forest and Odenwald highlands and is found intermittently across the study area. The Keuper deposits are Upper Triassic sandstones and mudstones that fill much of this region. Major rivers in this area, the Neckar and the Enz and their tributaries, cut though the overlying loess and the underlying Keuper beds, until they reach the hard limestones of the Middle Triassic Muschelkalk.

This area has been studied in some detail in terms of strontium isotopes. ^87^Sr/^86^Sr values for the older higher granites, gneiss, and Buntsandstein of the Black Forest and Odenwald are generally in the range from 0.7095 to 0.7150 and even higher. The Alpine Forelands to the south of the Danube are expected to be comparable to Tertiary deposits, ranging between 0.7077 and 0.7095 [80, 81]. The Jurassic deposits of the Schwabian uplands should exhibit ratios ca. 0.7068 to 0.7079 [80, 82]. The Sr-isotope composition of the Triassic Keuper and Muschelkalk in the central region of the study area is reported as ranging between 0.7076 and 0.7080 [83].

There are a series of archaeological studies that provide bioavailable values for the study region. The most detailed investigation can be found in Knipper [79]. Numerous baseline samples were measured and mapped over large parts of southwest Germany to provide a very good view of variation in strontium isotope ratios across this region. In sum, a general distinction can be made in biologically available ^87^Sr/^86^Sr between the upland and the lowlands of southwest Germany, ranging between 0.7086 and 0.7103 in the sedimentary lowlands and river valleys, and from 0.710 to as high as 0.722 in the crystalline uplands of the Odenwald and the Black Forest.

#### Bavaria

**Fig 10:**
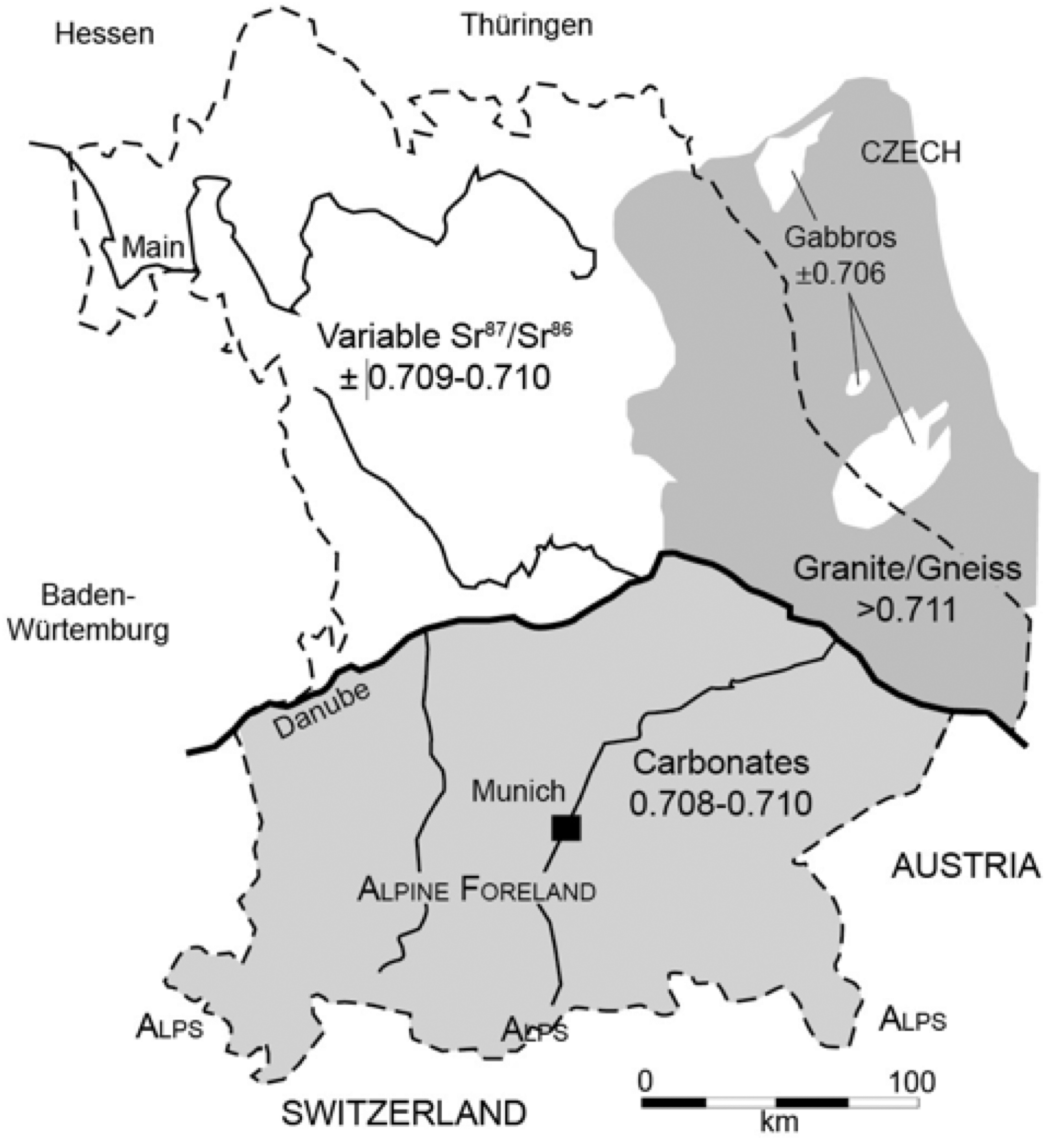
Strontium isotope geology of Bavaria (dotted line). Three major zones are indicated, carbonates in the south; granite and gneiss northeast of the Danube, and a varied geological landscape with mixed ^87^Sr/^86^Sr values to the northwest of the Danube. Redrawn from Schweissing and Grupe [87].

The geology of Bavaria can be divided into three major zones (Fig 10). North and east of the Danube are major deposits of old granite and gneiss with high ^87^Sr/^86^Sr values greater than 0.711. These uplands, known as the Bavarian and Oberpfälzer Forests, are the borderlands of the Carpathians. There are also several small pockets of gabbros and metagabbros (an intrusive igneous rock chemically similar to basalt) within this zone characterized by low ^87^Sr/^86^Sr values around 0.706. A second zone to the northwest contains lowlands and the Franconian and Swabian Jura and form a cuesta landscape. The geology of the region is heterogeneous including some loess deposits with varied ^87^Sr/^86^Sr values likely between 0.7086 and 0.7103 [84]. The fluvial deposits of the Danube valley are a mixture of the geologies of central Europe and exhibit a consistent ^87^Sr/^86^Sr value around 0.709 [85]. This value matches well with measured ^87^Sr/^86^Sr in the waters of the Danube where researchers find consistent ratios around 0.709 [86].

South of the Danube the region known as the Alpine Foreland grades into the Alps along the border with Switzerland and Austria. The Alps themselves are composed primarily of carbonates and were the source for gravel and other erosion products deposited under marine and freshwater conditions in the Alpine Foreland of southern Bavaria. There are a few reported values from Switzerland ranging from ca. 0.708 in Zürich [88] to between 0.708 and 0.709 for the majority of samples at Sion in the Rhone Valley in the western part of the country [89]. The Alpine Foreland is covered in many places with moraine and loess. Bioavailable values for the loess should range between 0.7086–0.7103 [84].

Investigation of Late Neolithic cemeteries along the Danube in Bavaria [70, 90, 91, 92, 93] documented the local strontium isotope ratios around 0.709. These values were confirmed by the analysis of four soil samples from Bell Beaker cemeteries in this area with a strontium isotope ratio between 0.7090 and 0.7099 with a mean of 0.7094. Berger et al. [94] reported on ^87^Sr/^86^Sr from a Roman fort just south of the Danube and observed a mean value of 0.7088 ± .0003 on enamel from a mule. Bickle et al. [95] report local human values from the Early Neolithic site of Aiterhofen, located south of the Danube on loess deposits. Approximately 60 of 64 measured individuals exhibited ^87^Sr/^86^Sr values between 0.709 and 0.710. Heyd et al. [96] reported ^87^Sr/^86^Sr from a series of Bell Beaker burials in southern Bavaria and found values ranging from 0.708 to 0.713. The majority of the samples (70/86 = 81%) fell between 0.708 and 0.710. The remainder (>0.711) were likely non-local individuals.

#### The Eurasian Steppe

In view of recent publications on the genetic composition of CW individuals, we feel it is warranted to include a discussion on the possibility that some of our non-locals may in fact be long distance movers from steppe areas. We will therefore summarize available baseline values for this region, to see whether it would be possible to distinguish long distance movers from this region to central Europe on the basis of Sr isotopes.

**Fig 11:**
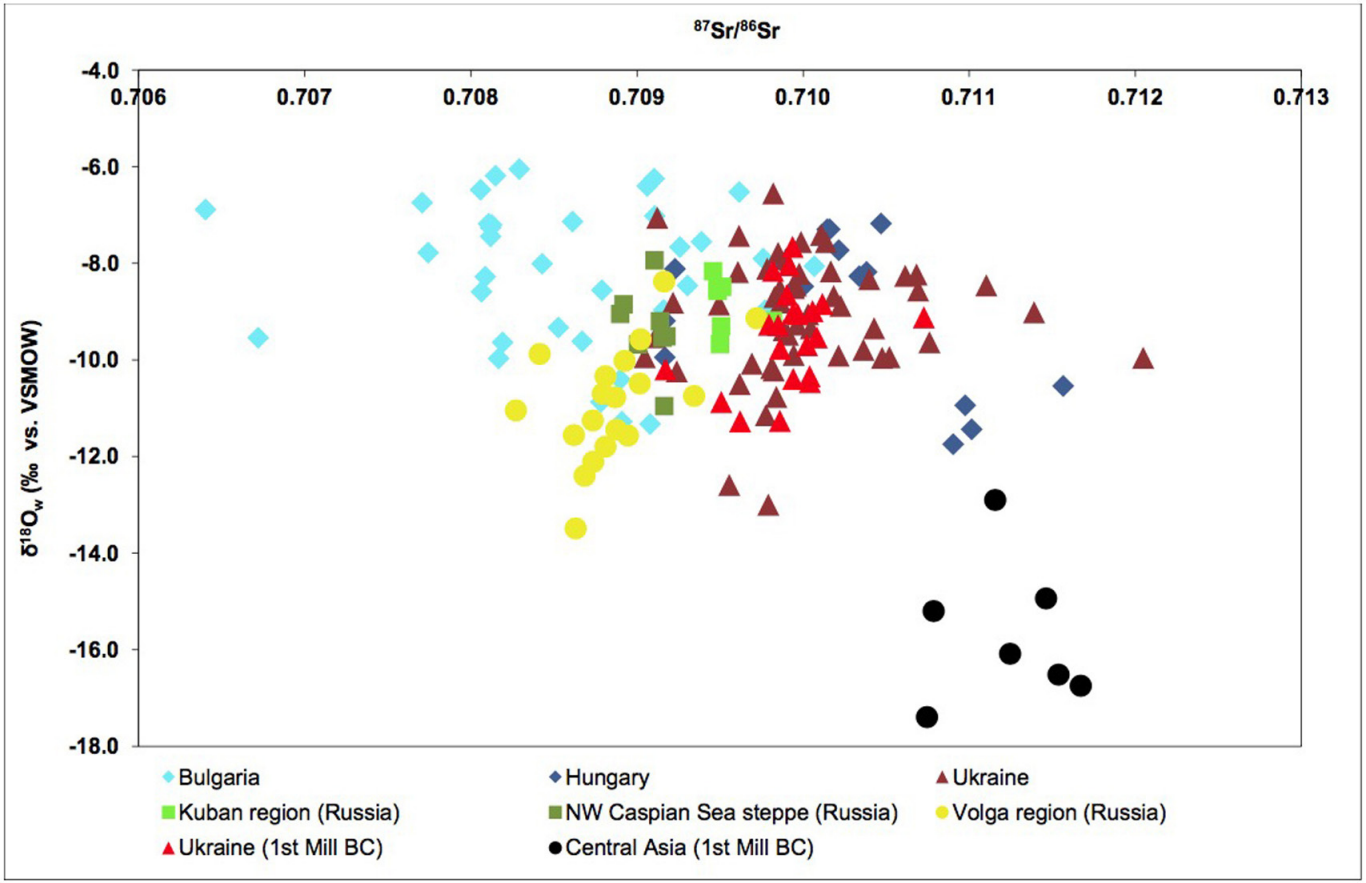
Gerling [97] scatterplot of ^87^Sr/^86^Sr and δ^18^O (VSMOW) from 142 human tooth enamel from across the steppe region. The vertical red line marks the 0.710 boundary in the ^87^Sr/^86^Sr values. Reprinted from [97] under a CC BY license, with permission from Claudia Gerling, original copyright 2014.

It is the case that the limited samples reported for the Eurasian steppe do not usually exhibit values above 0.711. Gerling [97, 98] reports a number of ^87^Sr/^86^Sr and δ18O values from the Eurasian steppe. She analyzed almost 400 samples from 27 sites across a vast area stretching from Bulgaria and Hungary in the Carpathian-Balkan region, Central and Eastern Ukraine in the North Pontic region, the Kuban region, the northwest Caspian steppes and the Middle and Lower Volga region in Russia, and the Altai Mountains in Kazakhstan. The two isotope ratios from 142 human tooth enamel samples from across the region are scatter plotted in Fig 11. The majority of the ^87^Sr/^86^Sr values fall between 0.708 and 0.710 and δ^18^O ranges between -6.0‰ and -12.0‰. Three areas had ^87^Sr/^86^Sr values greater than 0.710. The Ukraine exhibited 87Sr/86Sr values between 0.709 and 0.711, while Hungary and Central Asia had reported ratios between 0.710 and 0.712. Another study [99] of the eastern Ukraine recorded ^87^Sr/^86^Sr values from 25 fossil bison teeth between 0.708 and 0.710. Giblin et al. [100] measured 60 samples of archaeological fauna from the Great Hungarian Plain and found values ranging 0.709–0.710. Higher values were found in the upland areas of the Carpathian Mountains. Bioavailable samples of several modern horse teeth from northern Kazakhstan exhibited ratios around 0.710 [101]. Thus it would appear that the entire region investigated by Gerling has a wide range of ^87^Sr/^86^Sr values present and that values between 0.708 and 0.710 are common. As this range largely overlaps with values from southern Germany, we conclude that it is not possible to identify migrants from the steppe on the basis of Sr isotopes only.

### Oxygen Isotopes in Enamel Apatite

Oxygen isotopes have been widely used as a proxy for temperature in many climate and environmental studies and vary geographically in surface water and rainfall [102]. The oxygen isotope ratio in the skeleton mimics that of body water, and ultimately of drinking water [103, 104, 105], which in turn predominantly reflects local rainfall. Water from food and atmospheric oxygen are minor, secondary sources. Oxygen has three isotopes, ^16^O (99.762%), ^17^O (0.038%), and ^18^O (0.2%), all of which are stable and non-radiogenic. Isotope ratios in rainfall are greatly affected by enrichment or depletion of the heavy ^18^O isotope relative to ^16^O in water due to evaporation and precipitation [102]. Factors affecting rainfall δ^18^O values are primarily geographical: latitude, elevation, amount of precipitation, and distance from the evaporation source (e.g., an ocean).

Most isotope measurements are reported as a ratio of one isotope to another, lighter and more common cousin. Both carbon and oxygen isotopes are commonly reported as the per mil difference (‰ or parts per thousand) in the ratio of ^δ13C^ to ^12^C and ^18^O to ^16^O respectively, between a sample and a standard. This value is designated as δ^13^C and δ^18^O respectively. For oxygen, this value can be measured in either carbonate (CO_3_)^-2^ or phosphate (PO_4_)^-3^ ions of enamel apatite. Phosphate and carbonate produce comparable results. Less sample is needed for carbonate, preparation is less demanding, and results between laboratories are more comparable [106, 107, 108]. Two standards have been used to calculate oxygen isotope ratios by researchers investigating either carbonate (PDB, PeeDee Belemnite) or hydrological systems (VSMOW, Vienna Standard Mean Ocean Water) [109]. We report carbonate δ^18^O values in apatite using PDB as a standard.

At the same time, there are several difficulties in the application of oxygen isotope ratios to proveniencing in addition to diagenesis [110, 111]. Many parts of the temperate and tropical regions of the world have similar or identical δ^18^O values, ranging broadly from approximately -2.0‰ to -8.0‰ so that finding distinctive differences among samples in these regions is difficult [112]. In addition, considerable variability may be expected among the teeth of a single individual or even within a single tooth [113, 114, 115]. Because of the high variation present, the application of oxygen isotope ratios for provenience studies must be done with caution and remains experimental.

**Fig 12:**
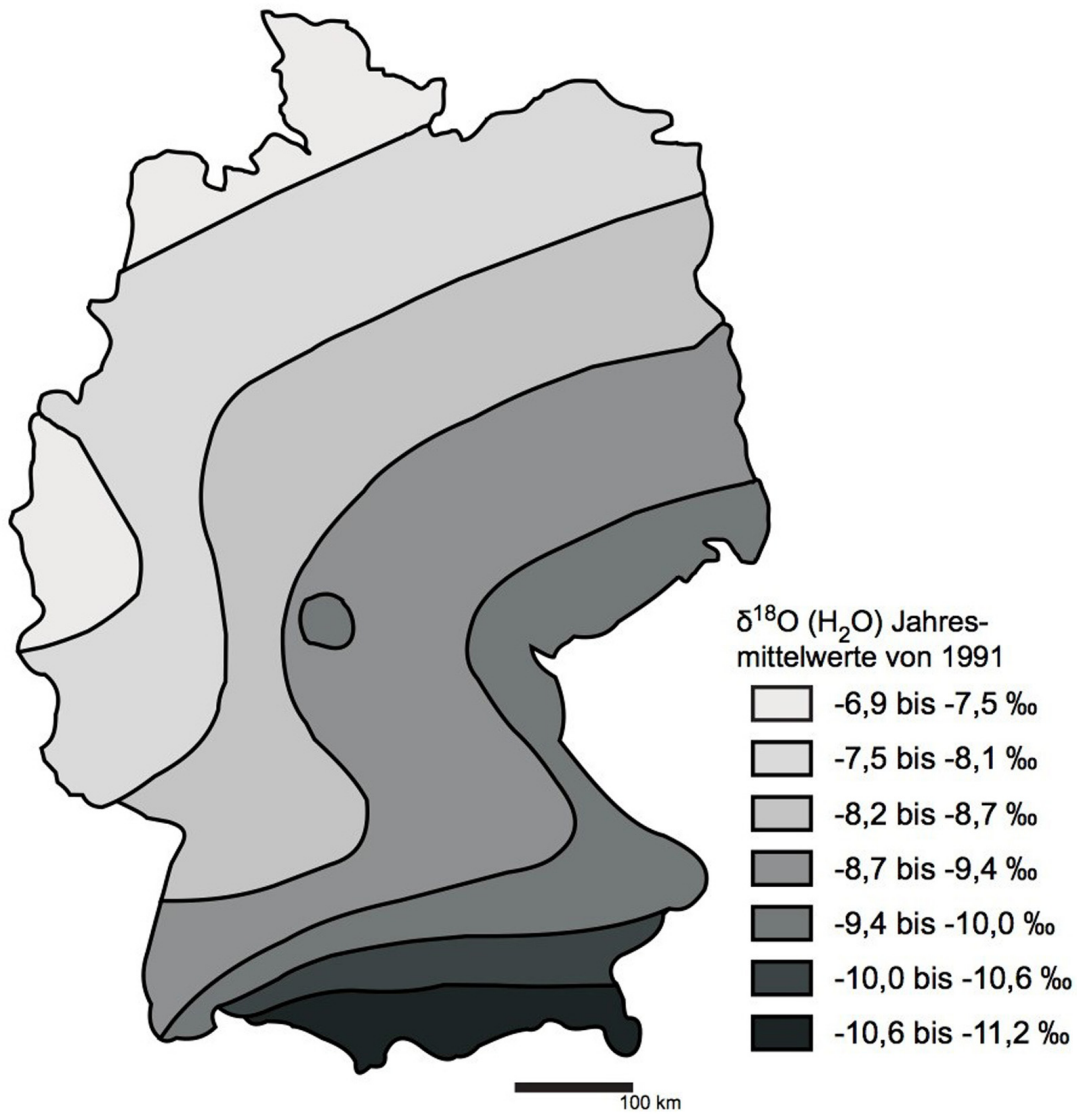
Isoscape map of mean annual δ18O values in Germany (VSMOW). Redrawn from Rozanski [116].

With regard to background values in southern Germany, there is detailed information available for oxygen isotope values (VSMOW) in modern precipitation [116, 117]. An isoscape map of δ^18^O values across Germany reveals a range of -6.9‰ to -11.2‰ largely varying from north to south with the most depleted values in the Bavarian Alps of southernmost Germany (Fig 12). The study area of southern Germany has values ranging from -8.7‰ to -11.2‰ across the region with the higher values with elevation in the Bavarian Alps and Alpine Foreland. To compare these values with our PDB data below we will note the direction of change in values as an indicator of potential places of origin.

### Strontium and Oxygen Isotope Results: Lauda-Königshofen

**Fig 13:**
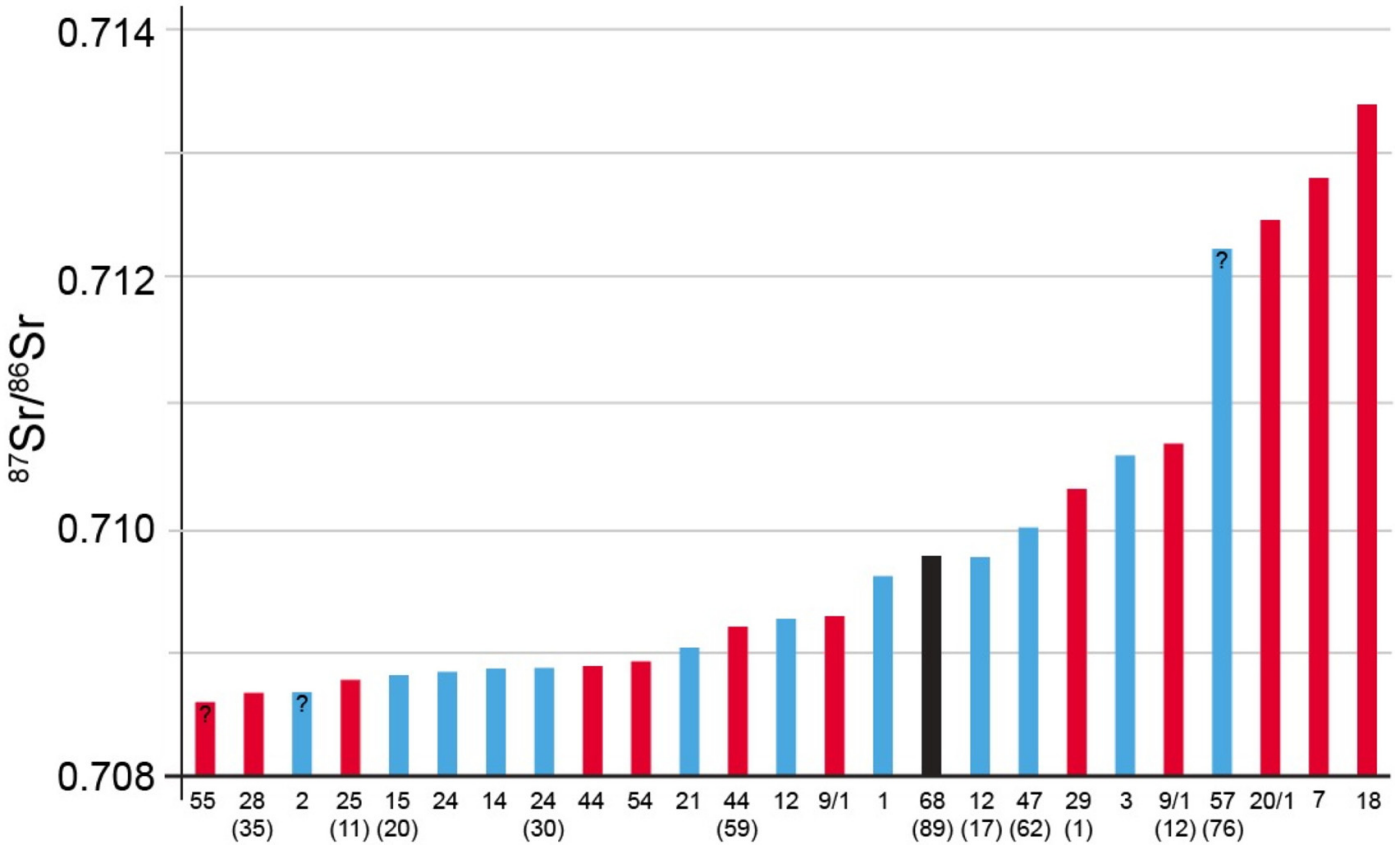
Bar graph of ranked ^87^Sr/^86^Sr from 25 individuals at Lauda-Königshofen. Red is female, blue is male, and black is unknown.

Information on the burials and the isotope ratios for strontium and oxygen for enamel powder are presented in S1 Table. The enamel samples from Lauda-Königshofen "Wöllerspfad" came from 25 individuals, of which 13 are males, 11 females, and 1 indeterminate. Data for nine of these individuals came from the earlier investigations [35, 36]. There are a total of 23 adults, 1 mature, and 1 juvenile individual reported here. The mean ^87^Sr/^86^Sr value was 0.7099 with 1 s.d. ±0.0014, with a minimum value of 0.7086 and a maximum of 0.7134. The ranked ^87^Sr/^86^Sr values for these samples are graphed in Fig 13. Bioavailable ^87^Sr/^86^Sr values in this region are expected to fall between 0.708 and 0.710 and seven of the individuals have values above 0.710. Four of these individuals, three females and one rather uncertain male, have values above 0.712, clearly non-local to the immediate area around Lauda-Königshofen. If we use the 0.710 value from bioavailable ^87^Sr/^86^Sr then 7 of the 25 individuals at Wöllerspfad are defined as non-local, ca. 28%.

**Fig 14:**
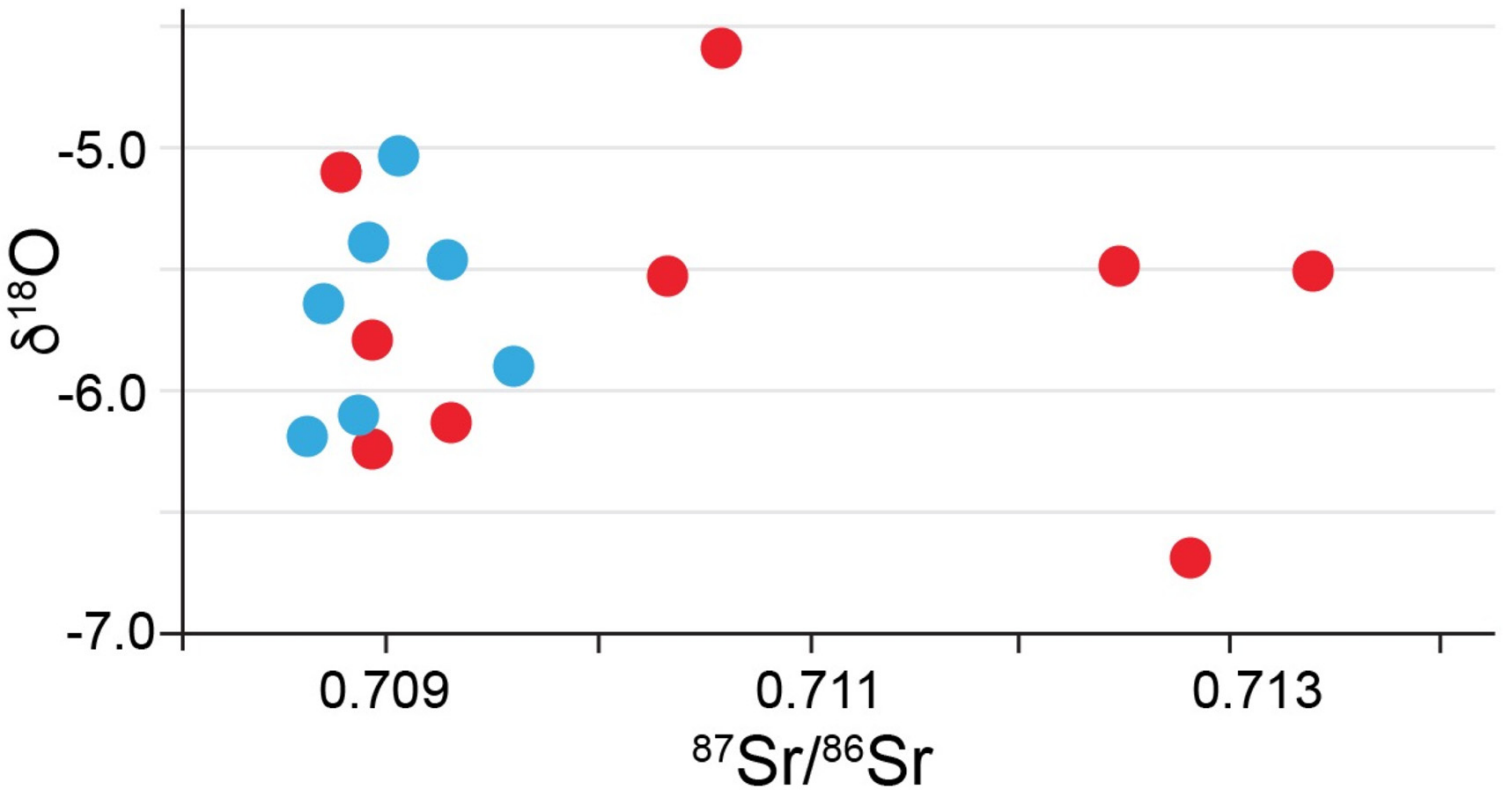
Scatterplot of enamel ^87^Sr/^86^Sr (x-axis) and δ^18^O from Lauda-Königshofen.

The average δ^18^O value for 16 samples (δ^18^O was not measured for the Menninger samples) was -5.2‰ with 1 s.d. of ±0.5, and a minimum value of -6.6‰ and a maximum value of -4.0‰. A scatterplot of ^87^Sr/^86^Sr vs. δ^18^O (Fig 14) emphasizes the two groups of ^87^Sr/^86^Sr values and the shared oxygen isotope ratios between the two groups. One of the three high ^87^Sr/^86^Sr individuals in this graph exhibits a very negative oxygen isotope ratio emphasizing her non-local origin. The other two individuals fall in the middle of the δ^18^O distribution.

### Strontium and Oxygen Isotope Results: Bergrheinfeld

The sample from Bergrheinfeld included a total of 19 individuals with 5 males, 9 females, and 5 indeterminate (4 infants and one juvenile). Information on the burials and the isotope ratios for strontium and oxygen for enamel powder are presented in S1 Table. The mean ^87^Sr/^86^Sr value was 0.7097 with 1 s.d. ±0.0009, with a minimum value of 0.7087 and a maximum of 0.7115. With the exception of the maximum values and greater variability at Lauda-Königshofen, these numbers are closely similar at the two sites.

**Fig 15:**
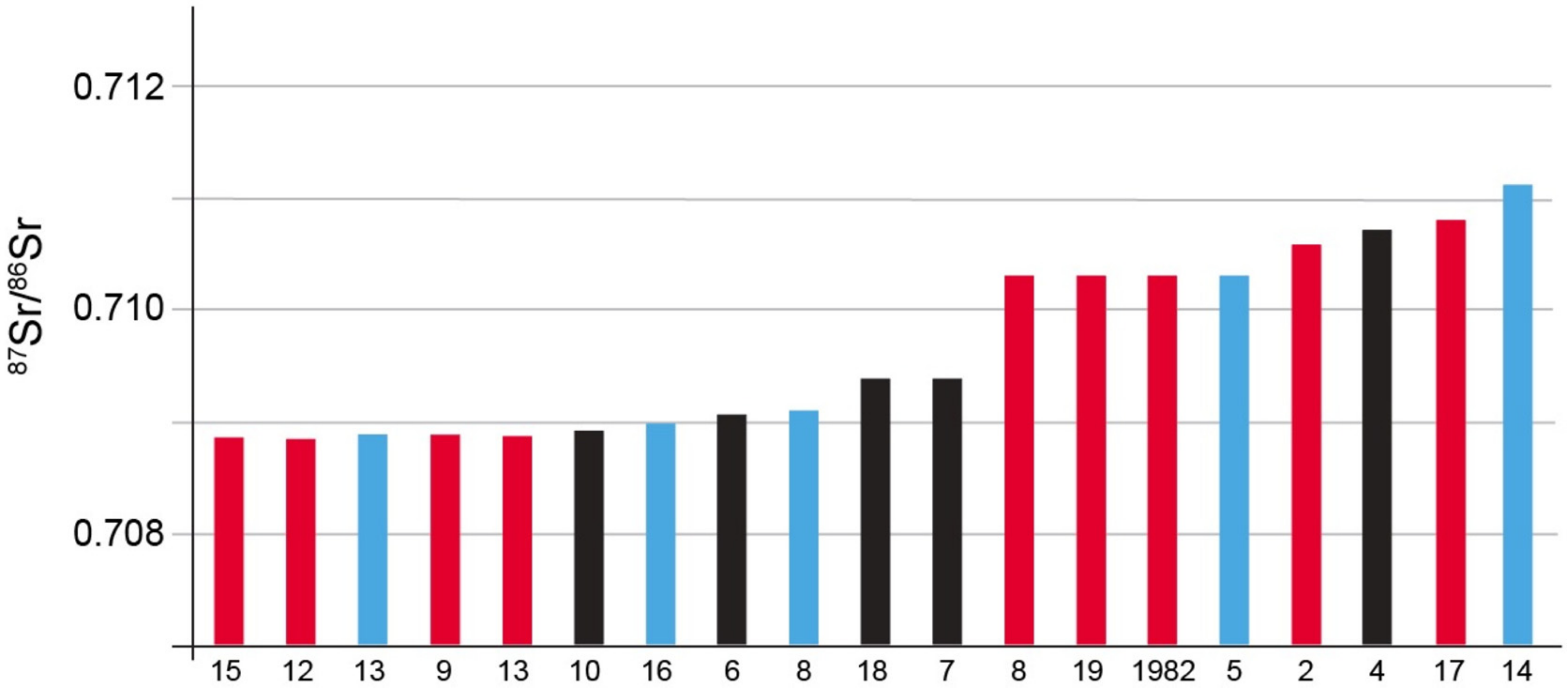
Bar graph of ranked ^87^Sr/^86^Sr from 19 individuals at Bergrheinfeld. Red is female, blue is male, and black is unknown.

The ranked ^87^Sr/^86^Sr values for these samples are graphed in Fig 15. There is a very clear break in the distribution of values with one group around 0.709 and a second group above 0.710. It is also notable that 5 of the 8 in the second, likely non-local group, are female while 2 are of uncertain sex.

**Fig 16:**
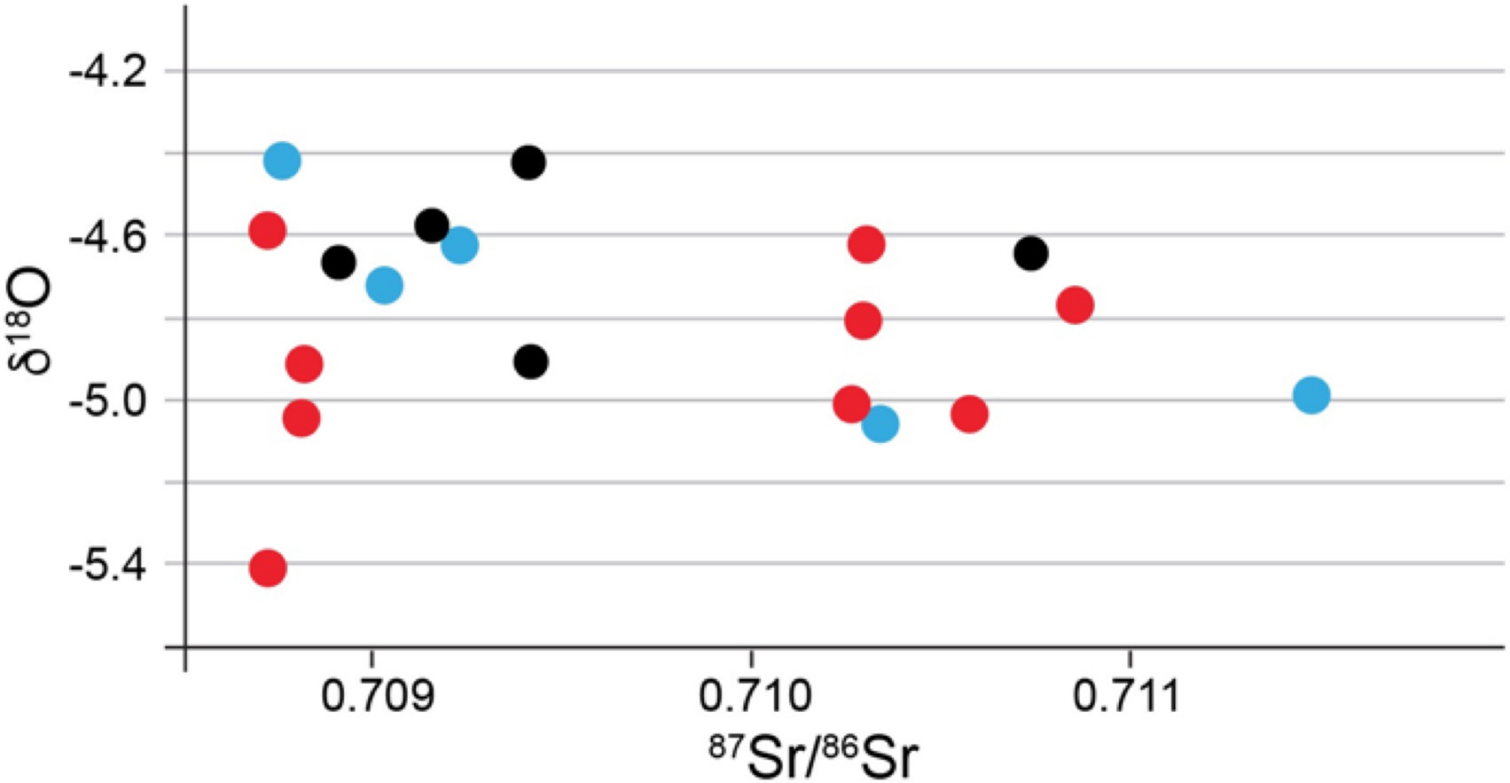
Scatterplot of enamel ^87^Sr/^86^Sr (x-axis) and δ^18^O from Bergrheinfeld.

The average δ^18^O value was -4.8‰ with 1 s.d. of ±0.25, and a minimum value of -5.1‰ and a maximum value of -4.4‰. A scatterplot of ^87^Sr/^86^Sr vs. δ^18^O (Fig 16) emphasizes the two groups of ^87^Sr/^86^Sr values and the shared oxygen isotope ratios between the two groups. The grave with the lowest δ^18^O value (-5.4‰, an adult female from grave 12) is also likely a non-local taken that it also has one of the lowest recorded Sr values.

**Fig 17:**
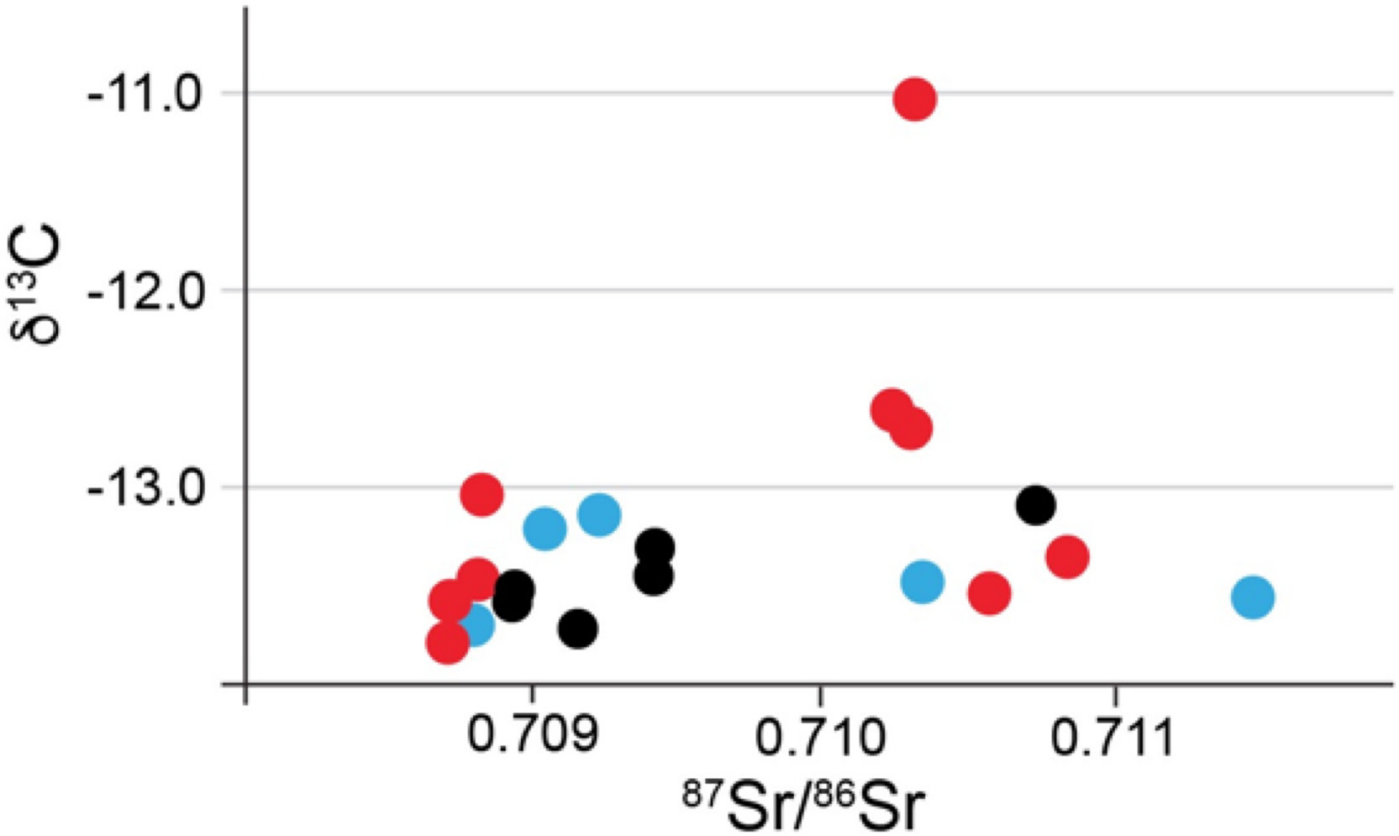
Scatterplot of enamel ^87^Sr/^86^Sr (x-axis) and δ^13^C from Bergrheinfeld.

A scatterplot of ^87^Sr/^86^Sr vs. δ^13^C (Fig 17) again reveals the two groups of strontium isotope ratios but also reveals a very distinctive individual in the higher strontium group with a very distinct δ^13^C value perhaps reflecting greater marine food intake or millet consumption. This individual, an adult female, may well be another non-local but from a location with similar ^87^Sr/^86^Sr values.

## Conclusions

The study of dietary isotopes reported here does not support a model of any dominant mode of subsistence. Instead, the picture is one of considerable variability at several levels; between sites as well as within them. In spite of the cultural homogeneity in the research area, we see a good deal of dietary variation. This variation correlates with sex and mobility, most clearly seen at Bergrheinfeld where non-local women seem to have a different diet from that of locals, and also from non-local males.

Compared to earlier Neolithic periods, there is a shift towards higher δ15N values, suggestive of a shift in diet and/or in cultivation practices. As noted above, there may be several different explanations for this shift, intense forms of cultivation, higher reliance on freshwater fish, on animal vs vegetable protein, or a greater reliance on milk and milk products. The latter is supported by a widespread opening of landscapes in some regions for grazing animals. Intense forms of cultivation and possibly manuring is also supported for the CW by weed composition and nitrogen isotopes.

In our data, it is clear from the diet evidence of non-local women that economies with more intense agriculture existed during the CW phase, creating an economic mosaic. To investigate whether this variability correlates with cultural background, however, demands more research.

Several observations can be made from the results of isotopic proveniencing. The proportion of non-local individuals is at least 7/25 (28%) at Lauda-Königshofen and 8/19 (42.1%) at Bergrheinfeld. Both of these proportions are quite high given the fact that the isotopic analysis of tooth enamel only identifies first generation mobility. If a cemetery contains several generations, these proportions are exceptionally high. Such high proportions identify a high degree of mobility among the individuals buried in the two CW cemeteries. Bayesian analysis of the radiocarbon dates from Bergrheinfeld, discussed above, suggests a use-life for the cemetery of less than 100 years, perhaps 3–4 generations. The high proportion of non-locals in the cemetery suggests that mobility continued across the generations. This is also supported by the ^14^C datings from the site.

The number and proportion of females with distinctive strontium isotope ratios is notable and suggests that women were more mobile than males in CW society. Such evidence fits well with recent genetic information documenting more varied haplogroups among CW females [14]. Müller et al. [2] suggest female exogamy as a means of maintaining lineage identify in the face of rapid, long-distance mobility. Haak et al. [25] also reported genetic and Sr isotope ratio differences between males and females at Eulau, Germany, suggesting female exogamy. The fact that such a difference is identifiable at all also suggests that males were largely stationary, at least in the sense that they were mostly born, raised and buried in the same locality. We suggest that this reflects a stable exogamic system where women moved to their husband’s settlements, existing at Bergrheinfeld for several generations. As no distinctions in burial treatment were associated with incoming women, either the exogamic exchange involved only CW groups, or incoming women were completely integrated into CW society.

Another issue concerns the possibility of long distance movement of the non-local individuals. Isotopic proveniencing is usually not successful at identifying homelands for non-locals because of the presence of multiple areas with similar isotopic signatures. In many cases it is sufficient to know that certain individuals were non-local or that mobility was pronounced in a society. On the other hand, some information regarding potential homelands might be available, particularly for the 3 high ^87^Sr/^86^Sr values in the females from Lauda-Königshofen. These values ranged between 0.712 and 0.714. There are of course various parts of Western and Central Europe where such values might be encountered, the nearest in Baden-Württemberg or eastern Bavaria. The steppe areas generally exhibit values lower than 0.712, as noted above.

One of the three high ^87^Sr/^86^Sr females from Lauda-Königshofen also had the most negative δ^18^O of the entire group, -6.2‰. More negative values generally point to the east in Eurasia as noted in both modern precipitation and Gerling’s data discussed above. The more negative oxygen value in this female may suggest her origins to the east. Otherwise it is difficult to suggest a specific homeland for the non-local individuals reported in the present study. These higher ^87^Sr/^86^Sr values are also present in the upland regions of southern Germany and may well represent less distant places of origins for the non-locals.

In sum, our study suggests that CW people in southern Germany specifically, and perhaps Central Europe as a whole, although genetically different, were similar in their economic practices to earlier Neolithic farming groups. CW groups appear to have consumed substantial amounts of both plants and animals in their diet.

At the same time, there is a clear shift in diet and/or cultivation practices, the nature of which remains to be explored.

Also, the substantial variation present at individual, local and regional levels is highlighted by this study. Such variability excludes any simplistic interpretation of CW economy as dominated by any single mode of subsistence. In combination with recent archaeological information for CW settlement and other studies of diet and mobility for this period, we would conclude that the CW people of southern Germany specifically, and perhaps Central Europe as a whole, continued largely in an agricultural way of life.

Although mobility was relatively high, it was not greatly different from earlier groups of farmers such as the Linearbandkeramik and the contemporary Bell Beaker folk of Western Europe in general and southern Germany in particular [69, 90, 93].

The correlation between sex, mobility and diet is one of the more interesting results of this study. We suggest that this may be interpreted as resulting from a stable system of female exogamy, involving various groups with different emphasis as regards subsistence economy. If some of these groups belonged to non-CW groups, as witnessed in Eulau, it was a clever long-term strategy for integrating such groups into the CW cultural network, and over time it may have led to increasing cultural homogeneity, but also economic variability.

## Supporting Information

S1 File Laboratory methods. (DOCX)

S1 Table Sample details and analysis results. (PDF)
