# Supplementary material for: Diet and Mobility in the Corded Ware of Central Europe
Source: PLoS One. 2016 May 25;11(5):e0155083. doi: 10.1371/journal.pone.0155083 (PMC4880197; doi:10.1371/journal.pone.0155083)
Supplement: S1 File — (DOCX) [file pone.0155083.s001.docx]

**S2. Methods**

No human or animal research involved. All necessary permits were obtained for the described study, which complied with all relevant regulations. Details on analysed samples are given in S1.

Collections from Lauda-Königshofen are held at Regierungspräsidium Stuttgart, Landesamt für Denkmalpflege, Arbeitsstelle Konstanz, Osteologie, Stromeyersdorfstraße 3, 78467 Konstanz. Samples and permissions were granted by Prof Dr Joachim Wahl.

Collections from all the Bavarian sites are held at Staatssammlung für Anthropologie und Paläoanatomie München, Karolinenplatz 2a, D-80333 München, Deutschland. Samples and permissions were granted by Dr. George McGlynn, Kurator, Stellv. Direktor.

Enamel Carbon and Oxygen samples

Teeth for δ13C and δ18O analysis were chemically cleaned using a standard procedure (Balasse et al. 2002). Enamel samples were placed in approximately 2 mL of 2-3% (v/v) solution of bleach for 8 hours and rinsed three times with deionized water, centrifuging the tubes between each aliquot. Then, 0.1 mL/mg of 0.1 M acetic acid was added to each tube for exactly 4 hours, and the samples were rinsed again with three aliquots of deionized water before being freeze-dried for analysis. Analysis of stable light isotopes was performed in the Environmental Isotope Laboratory (Department of Geosciences, University of Arizona) using a Kiel device attached to a Finnigan MAT252 ratio mass spectrometer. Samples are converted to CO2 with dehydrated 70°C phosphoric acid. External precision, as calculated from repeated measurements of standard reference materials (NBS-18 & NBS-19) is ±0.08‰ for δ13C and ±0.1‰ for δ18O.

Strontium samples

Teeth are cleaned with a dental drill equipped with a carbide burr (to remove any visible dirt or contamination. A sample is then taken from the tooth crown using a dental drill equipped with a circular saw. Approximately one quarter to one half of the tooth crown is removed. Remaining dentin is removed from the tooth fragment using a dental drill with a carbide bur, leaving the blue-white enamel. In some cases, fragments of enamel are removed from the base of the crown. Enamel is then ground to powder, weighed, and placed in a labeled plastic vial. Measurement of strontium isotopes was done at the Geochronology and Isotope Geochemistry Lab at the University of North Carolina-Chapel Hill by Paul Fullagar. Samples were dissolved in nitric acid and the strontium fraction purified by ion selective chromatography (Eichrom Sr resin), prior to analysis by TIMS on a VG Sector 54 mass spectrometer run in dynamic mode. Internal precision in the laboratory is consistently around 0.0007% standard error (or 1σ=0.00006 in the ratio of a particular sample). Long-term, repeated measurements of SRM-987 are around 0.710260—an acceptable difference from the recognized value of 0.710250—and raw sample values from individual runs are standardized to the recognized value of SRM-987.

Reference

Balasse M, Stanley HA, Smith AB, Price TD, The Seasonal Mobility Model for Prehistoric Herders in the South-western Cape of South Africa Assessed by Isotopic Analysis of Sheep Tooth Enamel. Journal of Archaeological Science 2002; 29: 917–932.
