# Supplementary material for: Diet and Mobility in the Corded Ware of Central Europe
Source: PLoS One. 2016 May 25;11(5):e0155083. doi: 10.1371/journal.pone.0155083 (PMC4880197; doi:10.1371/journal.pone.0155083)
Supplement: S1 Table — (PDF) [file pone.0155083.s002.pdf]

S1 Analysis results

Sitename	RISE#	Context	Labno	bp uncal	2Sigma	d13Ccoll	d15Ncoll	d13Cenam	d13C spacing	Sr86/Sr87	d18Oenam	Region	Sex	Ageclass
Altdorf	RISE437		OxA-32090	3993	29	-20.7	9.9	-14.6	6.1	0.7126	-5.0	Bavaria	F	Adult
Altdorf			Erl-7541	3941	48							Bavaria	F	Adult
Bergrheinfeld	RISE441	burial 16	OxA-31805	4043	27	-19.5	11.1	-13.2	6.3	0.7090	-4.7	Bavaria	M	juv
Bergrheinfeld	RISE442	burial 17	OxA-31806	4047	27	-20.6	10.3	-13.4	7.3	0.7108	-4.8	Bavaria	F	Adult
Bergrheinfeld	RISE443	adult female 1982	OxA-31807	3992	28	-19.5	9.7	-11.0	8.5	0.7103	-4.8	Bavaria	F	Adult
Bergrheinfeld	RISE444	burial 19	OxA-31894	4048	27	-19.6	9.8	-12.7	6.9	0.7103	-4.6	Bavaria	F	juv
Bergrheinfeld	RISE445	burial 7	OxA-31879	4047	27	-19.2	10	-13.3	5.9	0.7094	-4.4	Bavaria	nd	inf II
Bergrheinfeld	RISE446	burial 13 male	UBA-27950	4015	38	-19.5	11.2	-13.7	5.8	0.7088	-4.4	Bavaria	M	Adult
Bergrheinfeld	RISE447	burial 8 female	OxA-31808	4025	28	-20.5	9.9	-12.6	7.9	0.7103	-5.0	Bavaria	F	Adult
Bergrheinfeld	RISE448	burial 12	OxA-31809	3996	28	-19.4	11.5	-13.6	5.8	0.7087	-5.4	Bavaria	F	Adult
Bergrheinfeld	RISE449	burial 8 male	OxA-31810	4015	28	-19.3	11.3	-13.1	6.2	0.7092	-4.6	Bavaria	M	Adult
Bergrheinfeld	RISE450	burial 14	OxA-31811	4050	28	-20.1	10	-13.5	6.6	0.7115	-5.0	Bavaria	M	juv
Bergrheinfeld	RISE451	burial 18	P-38111					-13.4		0.7094	-4.9	Bavaria	nd	inf II
Bergrheinfeld	RISE452	burial 10	OxA-31947	4080	33	-19.7	11.3	-13.6	6.2	0.7089	-4.7	Bavaria	nd	inf II
Bergrheinfeld	RISE453	burial 5	OxA-31948	4016	31	-19.6	11	-13.5	6.1	0.7103	-5.1	Bavaria	M	Adult
Bergrheinfeld	RISE454	burial 9	OxA-31949	3994	32	-19.5	11.1	-13.5	6.0	0.7088	-5.0	Bavaria	F	Adult
Bergrheinfeld	RISE455	burial 13 female	OxA-31950	4009	31	-19.7	11.4	-13.0	6.7	0.7088	-4.9	Bavaria	F	Adult
Bergrheinfeld	RISE456	burial 15	OxA-31951	3971	31	-19.3	10.5	-13.7	5.6	0.7087	-4.6	Bavaria	F	Adult
Bergrheinfeld	RISE457	burial 2	OxA-31952	4003	32	-20.1	10.6	-13.5	6.6	0.7106	-5.0	Bavaria	F	Adult
Bergrheinfeld	RISE458	burial 4	OxA-31988	3979	30	-19.3	9.2	-13.1	6.2	0.7107	-4.6	Bavaria	nd	juv
Bergrheinfeld	RISE459	burial 6	OxA-31989	4064	30	-19.3	10.4	-13.7	5.6	0.7091	-4.6	Bavaria	nd	inf II
Kelheim	RISE438	burial 22	OxA-32091	4143	29	-20.6	10.2	-17.1	3.5	0.7169	-4.1	Bavaria	F	Adult
Kelheim		burial 22	Hv-8535	4175	70							Bavaria	F	Adult
Lauda-Königshofen	RISE327	Grave 1						-13.1		0.7096	-5.4	Baden-Württemberg	M?	Adult
Lauda-Königshofen	RISE328	Grave 2						-12.2		0.7087	-5.2	Baden-Württemberg	M?	Adult
Lauda-Königshofen	RISE329	Grave 3						-13.8		0.7106	-4.0	Baden-Württemberg	M	Adult
Lauda-Königshofen	RISE330	Grave 7						-14.3		0.7128	-6.2	Baden-Württemberg	F	Adult
Lauda-Königshofen	RISE331	Grave 9/I	OxA-31175	4005	32	-19.6447	11.2777	-13.5	6.2	0.7093	-5.6	Baden-Württemberg	F	Adult
Lauda-Königshofen	RISE332	Grave 12						-12.7		0.7093	-5.0	Baden-Württemberg	M	Adult
Lauda-Königshofen	RISE333	Grave 14	OxA-31062	4032	30	-19.7932	11.7795	-10.1	9.7	0.7089	-4.9	Baden-Württemberg	M	Adult
Lauda-Königshofen	RISE334	Grave 18	P-39960					-14.2		0.7134	-5.0	Baden-Württemberg	F	Adult
Lauda-Königshofen	RISE335	Grave 20/I	OxA-31063	4004	29	-19.7212	11.0148	-13.4	6.3	0.7125	-5.0	Baden-Württemberg	F	Adult
Lauda-Königshofen	RISE336	Grave 21	OxA-31064	3965	31	-19.7317	11.1992	-13.8	5.9	0.7090	-4.5	Baden-Württemberg	M	Adult
Lauda-Königshofen	RISE337	Grave 24	OxA-31065	4023	31	-19.6074	11.4933	-13.7	5.9	0.7088	-5.6	Baden-Württemberg	M	Adult
Lauda-Königshofen	RISE338	Grave 25/II	OxA-31066	4001	33	-19.7436	11.5307	-13.4	6.4	0.7088	-4.6	Baden-Württemberg	F	Adult
Lauda-Königshofen	RISE339	Grave 29/I						-13.3		0.7103	-5.0	Baden-Württemberg	F	Adult
Lauda-Königshofen	RISE340	Grave 44						-12.8		0.7089	-5.7	Baden-Württemberg	F	Adult
Lauda-Königshofen	RISE341	Grave 47										Baden-Württemberg	M	Mat
Lauda-Königshofen	RISE342	Grave 49/I										Baden-Württemberg	M	Adult
Lauda-Königshofen	RISE343	Grave 54	OxA-31067	4013	30	-20.032	11.9889	-9.5	10.5	0.7089	-5.3	Baden-Württemberg	F	Adult
Lauda-Königshofen	RISE344	Grave 55	P-39968					-12.9		0.7086	-5.7	Baden-Württemberg	F?	juv
Lauda-Königshofen		Grave 9/I, ind 12								0.7107		Baden-Württemberg	F	Adult
Lauda-Königshofen		Grave 12, ind 17								0.7098		Baden-Württemberg	M	Adult
Lauda-Königshofen		grave 15, ind 20								0.7088		Baden-Württemberg	M	Adult
Lauda-Königshofen		grave 24, ind 30								0.7089		Baden-Württemberg	M	Adult
Lauda-Königshofen		grave 28, ind 35								0.7087		Baden-Württemberg	F	Adult
Lauda-Königshofen		grave 44, ind 59								0.7092		Baden-Württemberg	F	Adult
Lauda-Königshofen		grave 47, ind 62								0.7100		Baden-Württemberg	M	Mat
Lauda-Königshofen		grave 57, ind 76								0.7122		Baden-Württemberg	M?	Adult
Lauda-Königshofen		Grave 68, ind 89								0.7098		Baden-Württemberg	nd	Adult
Poldering	RISE439		OxA-32092	4120	29	-21	9.8	-15.3	5.7	0.7108	-4.8	Bavaria	nd	Adult?
Tiefbrunn	RISE434	3/1	UBA-27946	4161	34	-21.4	11.3	-14.6	6.8	0.7105	-5.9	Bavaria	M	Adult
Tiefbrunn	RISE435	3/2	UBA-27947	4094	33	-21.3	11.2	-14.4	6.9	0.7103	-4.5	Bavaria	F	Inf
Tiefbrunn	RISE436	3/3	UBA-27948	4124	31	-21.5	10.8	-14.4	7.1	0.7116	-5.3	Bavaria	M	Adult
Wolkshausen	RISE440	burial 9	UBA-27949	3968	33	-19.9	11.9	-14.6	5.3	0.7102	-4.4	Bavaria	M	Adult
